# Genomic analysis of lean individuals with NAFLD identifies monogenic disorders in a prospective cohort study

**DOI:** 10.1016/j.jhepr.2023.100692

**Published:** 2023-02-02

**Authors:** Melanie Zheng, Daniel Q. Huang, Chigoziri Konkwo, Saaket Agrawal, Amit V. Khera, Rohit Loomba, Sílvia Vilarinho, Veeral Ajmera

**Affiliations:** 1 Departments of Internal Medicine, Section of Digestive Diseases, and of Pathology, Yale School of Medicine, New Haven, CT, USA; 2 NAFLD Research Center, Division of Gastroenterology, University of California at San Diego, La Jolla, CA, USA; 3 Department of Medicine, Yong Loo Lin School of Medicine, National University of Singapore, Singapore; 4 Program in Medical and Population Genetics, Broad Institute of MIT and Harvard, Cambridge, MA, USA; 5 Verve Therapeutics, Cambridge, MA, USA; 6 Department of Pathology, Yale School of Medicine, New Haven, CT, USA

**Keywords:** NAFLD, Non-obese, Rare genetic variants, Whole exome sequencing, APOB, apolipoprotein B, ALT, alanine aminotransferase, FHBL, familial hypobetalipoproteinaemia, LOFHC, high-confidence predicted loss-of-function, MRE, magnetic resonance elastography, MRI, magnetic resonance imaging, MRI-PDFF, magnetic resonance imaging proton density fat fraction, NAFLD, non-alcoholic fatty liver disease, UCSD, University of California San Diego, WES, whole exome sequencing

## Abstract

**Background & Aims:**

Lean patients with non-alcoholic fatty liver disease (NAFLD) represent 10–20% of the affected population and may have heterogeneous drivers of disease. We have recently proposed the evaluation of patients with lean NAFLD without visceral adiposity for rare monogenic drivers of disease. Here, we aimed to validate this framework in a well-characterised cohort of patients with biopsy-proven NAFLD by performing whole exome sequencing.

**Methods:**

This prospective study included 124 patients with biopsy-proven NAFLD and paired liver biopsies who underwent standardised research visits including advanced magnetic resonance imaging (MRI) assessment of liver fat and stiffness.

**Results:**

Six patients with lean NAFLD were identified and underwent whole exome sequencing. Two lean patients (33%) were identified to have monogenic disorders. The lean patients with monogenic disorders had similar age, and anthropometric and MRI characteristics to lean patients without a monogenic disorder. Patient 1 harbours a rare homozygous pathogenic mutation in *ALDOB* (aldolase B) and was diagnosed with hereditary fructose intolerance. Patient 2 harbours a rare heterozygous mutation in apolipoprotein B (*APOB*). The pathogenicity of this *APOB* variant (p.Val1856CysfsTer2) was further validated in the UK Biobank and associated with lower circulating APOB levels (beta = -0.51 g/L, 95% CI -0.65 to -0.36 g/L, *p* = 1.4 × 10^-11^) and higher liver fat on MRI (beta = +10.4%, 95% CI 4.3–16.5%, *p* = 8.8 × 10^-4^). Hence, patient 2 was diagnosed with heterozygous familial hypobetalipoproteinaemia.

**Conclusions:**

In this cohort of well-characterised patients with lean NAFLD without visceral adiposity, 33% (2/6) had rare monogenic drivers of disease, highlighting the importance of genomic analysis in this NAFLD subtype.

**Impact and Implications:**

Although most people with non-alcoholic fatty liver disease (NAFLD) are overweight or obese, a subset are lean and may have unique genetic mutations that cause their fatty liver disease. We show that 33% of study participants with NAFLD who were lean harboured unique mutations that cause their fatty liver, and that these mutations had effects beyond the liver. This study demonstrates the value of genetic assessment of NAFLD in lean individuals to identify distinct subtypes of disease.

## Highlights

• In a well-characterized cohort of patients with biopsy-proven NAFLD, 6 out of 124 (5%) were lean without visceral adiposity.
• Whole-exome sequencing uncovered monogenic causes of fatty liver disease in 2 of 6 lean patients (33%):
• A mutation in *APOB* causing heterozygous familial hypobetalipoproteinemia and a mutation in *ALDOB* causing hereditary fructose intolerance.
• Pathogenicity of the detected *APOB* variant (p.Val1856CysfsTer2) was validated in the UK Biobank.
• High rate of monogenic disorders in lean patients highlights the importance of genetic assessment in this NAFLD subtype.

## Graphical abstract

**Figure:**
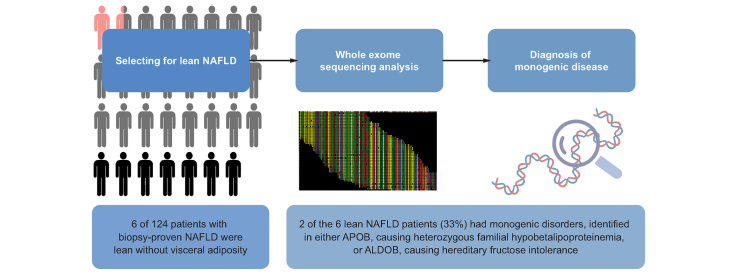

## Introduction

The prevalence of non-alcoholic fatty liver disease (NAFLD) continues to grow, affecting an estimated 60–80 million people in the United States, and a subset of patients will progress to advanced liver disease including cirrhosis and hepatocellular carcinoma.^1^ Patients with NAFLD who are lean represent 10–20% of the total population, and observational studies have yielded conflicting results with regard to disease severity and prognosis,^2^^,^^3^ which may be related to more heterogeneous drivers of disease.

A previous study demonstrating the clinical utility of genomic analysis in the diagnosis and management of adult patients with liver disease of unknown aetiology revealed previously unappreciated monogenic disorders in three non-obese patients with NAFLD.^4^ This finding led us to propose a framework for genomic evaluation of lean patients with NAFLD who lack visceral adiposity, to identify rare genetic variants that may have therapeutic implications and elucidate additional pathogenic mechanisms.^5^ Here, we performed whole exome sequencing (WES) on lean individuals from a well-phenotyped longitudinal cohort with biopsy-proven NAFLD to evaluate for rare, monogenic drivers of disease.

## Patients and methods

This is a longitudinal study derived from a well-characterised prospective cohort of patients with biopsy-proven NAFLD and paired liver biopsies who underwent a standard research visit that included history, physical examination, biochemical testing, and paired liver biopsy assessment (using the Non-alcoholic Steatohepatitis Clinical Research Network histologic scoring system) at the University of California San Diego (UCSD) NAFLD Research Center from 2006 through 2019. All patients provided written informed consent before enrolling in the study, and the study was approved by the UCSD Institutional Review Board. At baseline, all patients underwent a standardised clinical evaluation including detailed history, anthropometric exam, and laboratory testing at the UCSD NAFLD Research Center. Patients ≥18 years of age with biopsy-proven NAFLD were included and were identified as lean NAFLD by BMI ≤ 25 kg/m^2^ for non-Asians and ≤ 23 kg/m^2^ for Asians. Germline DNA was extracted from blood samples using standard methods. Germline DNA was captured using xGen exome V2 exome enrichment probes (Integrated DNA Technologies Coralville, Iowa) and sequenced using the Illumina NovaSeq platform (San Diego, California). The apolipoprotein B (*APOB*) rare variant validation was performed in the UK Biobank. All analyses were performed using R 3.6.0 (R Foundation for Statistical Computing, Vienna, Austria). Additional details are provided in the Supplementary information.

## Results

Of 124 participants with biopsy-proven NAFLD who had longitudinal follow-up,^6^ six patients with lean NAFLD, defined as a BMI ≤ 25 kg/m^2^ for non-Asians and ≤ 23 kg/m^2^ for Asians, were identified. Lean and non-lean participants were similar with regard to age, sex, diabetes status, laboratory parameters, liver histology, and magnetic resonance imaging proton density fat fraction (MRI-PDFF) and magnetic resonance elastography (MRE) (Table S1). There was no difference in longitudinal change in histology, MRI-PDFF, or MRE between patients with lean NAFLD and those with non-lean NAFLD (Table S2). Six participants with lean NAFLD underwent whole exome sequencing (WES) of germline DNA (Table S3). Using the WES analysis pipeline (Fig. S1), we identified a monogenic disorder in two of these adult lean individuals with NAFLD (Table S4). Lean patients with monogenic disorders were of similar age and BMI and had similar fasting insulin levels to lean patients without an identified monogenic disorder (Table 1). None of the patients in this study with biopsy-proven lean NAFLD harboured the protective rare variant in cell death-inducing DFFA-like effector B.

**Table 1:** **Baseline characteristics of participants with lean NAFLD, stratified by presence of pathogenic mutations**.

	All lean patients (n = 6)	No monogenic mutations (n = 4)	Monogenic mutations (n = 2)	*p* value
**Demographic profile**				

Age (years)	59.50 [53.75, 63.00]	59.50 [51.25, 63.00]	61.00 [57.00, 65.00]	0.639
Female, n (%)	5 (83.3)	3 (75.0)	2 (100.0)	1
BMI (kg/m^2^)	23.42 [21.74, 24.54]	23.42 [22.43, 24.23]	23.13 [22.22, 24.04]	1
Diabetes mellitus, n (%)	3 (50.0)	3 (75.0)	0 (0.0)	0.4
Hispanic, n (%)	1 (16.7)	1 (25.0)	0 (0.0)	1

**Biochemical data**				

AST (U/L)	41.50 [36.50, 72.75]	38.00 [32.75, 59.00]	62.50 [52.25, 72.75]	0.355
ALT (U/L)	45.50 [43.25, 120.50]	45.00 [41.75, 91.50]	94.50 [69.25, 119.75]	0.643
HbA1c (%)	5.95 [5.67, 6.15]	5.80 [5.60, 6.05]	6.45 [6.18, 6.72]	0.348
Total bilirubin (mg/dl)	0.50 [0.35, 0.65]	0.50 [0.30, 0.70]	0.50 [0.50, 0.50]	1
Direct bilirubin (mg/dl)	0.15 [0.10, 0.20]	0.15 [0.10, 0.19]	0.15 [0.12, 0.17]	0.812
INR	1.05 [1.00, 1.10]	1.10 [1.08, 1.10]	1.00 [1.00, 1.00]	0.114
Albumin (g/dl)	4.70 [4.55, 4.77]	4.60 [4.47, 4.78]	4.75 [4.73, 4.77]	0.481
Total cholesterol (mg/dl)	188.00 [167.00, 215.00]	188.00 [171.00, 212.50]	160.50 [131.25, 189.75]	0.643
HDL (mg/dl)	46.00 [40.50, 56.75]	45.00 [38.25, 52.25]	57.00 [49.50, 64.50]	0.355
LDL (mg/dl)	110.00 [90.25, 120.75]	110.00 [96.75, 127.75]	84.00 [65.50, 102.50]	0.643
TG (mg/dl)	149.50 [103.50, 191.00]	183.00 [148.75, 211.50]	99.00 [82.50, 115.50]	0.165
Insulin	13.00 [8.00, 23.00]	13.00 [10.50, 25.50]	14.50 [10.25, 18.75]	0.564
Interval between biopsies (months)	17.35 [13.53, 24.18]	13.65 [11.48, 15.62]	30.75 [28.03, 33.48]	0.064

**Liver histology findings baseline**				

NAS	5.50 [4.25, 6.00]	4.50 [3.75, 5.25]	6.00 [6.00, 6.00]	0.14
Fibrosis stage, n (%)				1
0	1 (16.7)	1 (25.0)	0 (0.0)	
1	1 (16.7)	1 (25.0)	0 (0.0)	
2	1 (16.7)	0 (0.0)	1 (50.0)	
3	2 (33.3)	1 (25.0)	1 (50.0)	
4	1 (16.7)	1 (25.0)	0 (0.0)	
Steatosis score, n (%)				0.467
0	0 (0.0)	0	0	
1	1 (16.7)	2 (50.0)	0 (0.0)	
2	3 (50.0)	2 (50.0)	2 (100.0)	
3	2 (33.3)	2 (50.0)	0 (0.0)	
Lobular inflammation score, n (%)				0.467
0	0	0	0	
1	2 (33.3)	2 (50.0)	0 (0.0)	
2	4 (66.7)	2 (50.0)	2 (100.0)	
3	0 (0.0)	0	0	
Ballooning score, n (%)				1
0	1 (16.7)	1 (25.0)	0 (0.0)	
1	3 (50.0)	2 (50.0)	1 (50.0)	
2	2 (33.3)	1 (25.0)	1 (50.0)	

**Imaging results**				

MRI-PDFF (%)	18.89 [14.57, 23.40]	18.89 [14.08, 23.46]	19.02 [16.51, 21.53]	1
MRE	3.03 [2.82, 3.70]	3.03 [2.69, 3.40]	3.34 [3.06, 3.61]	1

Median values are provided with IQR in brackets, unless otherwise noted as n (%). Categorical variables tested using the Fisher exact test. Continuous variables compared using the *t* test or Wilcoxon’s two-sample test, as appropriate. ALT, alanine transaminase; AST, aspartate transaminase; HbA_1c_, haemoglobin A_1c_; INR, international normalised ratio; MRE, magnetic resonance elastography; MRI-PDFF, magnetic resonance imaging proton density fat fraction; NAS, NAFLD activity score; TG, triglycerides.

### Diagnosis of hereditary fructose intolerance in patient 1

Patient 1 had a BMI of 21.3 kg/m^2^, with biopsy-proven non-alcoholic steatohepatitis with stage 2 fibrosis and MRI-PDFF of 24% consistent with severe steatosis. She was found to harbour a rare homozygous missense variant (chr9:104189856; C>G; p.Ala150Pro) in *ALDOB*, which encodes aldolase B. Biallelic variants in this gene cause hereditary fructose intolerance. Aldolase B is the enzyme responsible for catalysing fructose 1,6-bisphosphate into glyceraldehyde 3-phosphate and dihydroxyacetone phosphate, and of fructose 1-phosphate into glyceraldehyde and dihydroxyacetone phosphate. Given the toxic metabolite accumulation as a result of the ingestion of fructose or sucrose, affected patients may present with hypoglycaemia, hepatic steatosis, and proximal renal tubulopathy.^7^ This variant was predicted to be damaging by the additional *in silico* prediction models MetaSVM, SIFT, and PolyPhen-2 and has been reported as pathogenic in the ClinVar National Center for Biotechnology Information database. Experimental studies have shown that this missense mutation reduces substrate affinity and enzyme stability and activity within aldolase B.^8^ This variant in homozygosity or compound heterozygosity has been described in individuals affected with hereditary fructose intolerance. This patient reported nausea, abdominal pain, and hypoglycaemia exacerbated by fruit intake consistent with hereditary fructose intolerance. The patient had no family history of hereditary fructose intolerance in her first-degree relatives.

### Diagnosis of FHBL in patient 2

**Fig. 1:**
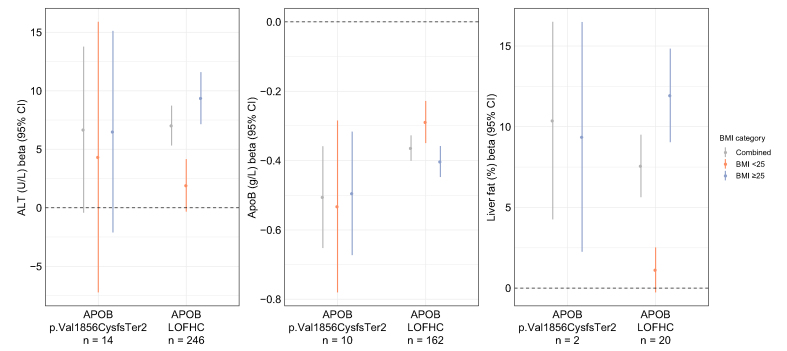
Effects of LOFHC variants in *APOB* on (A) ALT (B) ApoB levels, and (C) liver fat % in the UK Biobank across BMI strata. Up to 176,177 participants were available for the ALT analysis, up to 175,341 for the ApoB analysis, and up to 18,610 for the liver fat % analysis following quality control and removal of related samples (see Patients and methods). Effect sizes and 95% CIs are reported separately for the p.Val1856CysfsTer2 variant and all other *APOB* LOFHC variants corresponding to linear regression models adjusted for age, sex, and the first 10 principal components of genetic ancestry (liver fat models were additionally adjusted for MRI serial number). We combined all *APOB* LOFHC variants including p.Val1856CysfsTer2 to test for a BMI interaction and noted a significant interaction with both ALT (*p* = 0.008) and liver fat % (*p* = 6.3 × 10^-5^). ALT, alanine aminotransferase; APOB, apolipoprotein B; liver fat %, image-derived liver fat percentage; LOFHC, high-confidence predicted loss-of-function; MRI, magnetic resonance imaging.

Patient 2 had a BMI of 24.96 kg/m^2^, with biopsy-proven non-alcoholic steatohepatitis with stage 3 fibrosis and MRI-PDFF of 14%. WES of germline DNA from patient 2 revealed a heterozygous frameshift variant (chr2:21234172, AAC>A; p.Val1856CysfsTer2) in *APOB*. APOB is the primary apolipoprotein of chylomicrons and VLDL, IDL, and LDL particles.^9^ Familial hypobetalipoproteinaemia (FHBL) presents with low circulating lipid levels and increased hepatic steatosis.^4^^,^^5^ This frameshift variant has been reported as pathogenic in the ClinVar National Center for Biotechnology Information (NCBI) database and previously associated with FHBL, but it has also been annotated as likely benign in the ClinVar NCBI database. Hence, we went back to the patient to perform genotype–phenotype correlation.^4^^,^^10^ In addition to hepatic steatosis, the patient had low circulating lipid levels (LDL = 47 mg/dl, total cholesterol = 102 mg/dl, and triglycerides = 66 mg/dl) The patient’s APOB level was evaluated and was low at 39 mg/dl. She had no family history of hypobetalipoproteinaemia in first-degree relatives. We next studied the first 200,643 exome-sequenced participants of the UK Biobank to better characterise the clinical significance of the p.Val1856CysfsTer2 variant in *APOB*.^11^ Following a previously described genetic and sample quality control pipeline,^12^ we identified 14 (0.007%) heterozygous carriers of p.Val1856CysfsTer2, two of whom returned for a follow-up imaging visit for MRI-derived liver fat measurement. Carriers of p.Val1856CysfsTer2 had lower APOB levels (beta = -0.51 g/L, 95% CI -0.65 to -0.36 g/L, *p* = 1.4 × 10^-11^), higher liver fat (beta = +10.4%, 95% CI 4.3–16.5%, *p* = 8.8 × 10^-4^), and a trend toward higher alanine aminotransferase (ALT) (beta =+6.7 U/L, 95% CI -0.4 to 13.8 U/L, *p* = 0.07). We next studied participants who carried loss-of-function transcript effect estimator (LOFTEE)-derived high-confidence predicted loss-of-function (LOFHC) variants in *APOB*, excluding p.Val1856CysfsTer2. We observed 280 heterozygote carriers of LOFHC variants in *APOB* (21 with liver imaging) across 104 variants, all with minor allele frequency less than 0.01%. Associations with ALT, APOB, and liver fat in these carriers were comparable with those of the p.Val1856CysfsTer2 variant (Fig. 1). In addition, UK Biobank participants with low serum APOB levels were more likely to harbour LOFHC variants in *APOB* and microsomal triglyceride transfer protein (MTTP) (Table S5). Finally, we evaluated the interaction between BMI and *APOB* variants combining p.Val1856CysfsTer2 with all other LOFHC variants in *APOB* and found a positive interaction term for both ALT (*p* = 0.008) and liver fat (*p* = 6.3 × 10^-5^), suggesting that higher BMI amplifies the impact of the studied rare *APOB* variants on pathologic liver traits. Altogether, genotype and phenotype findings, as well as external validation of the impact of this rare variant, support that this patient has autosomal dominant *APOB*-related FHBL.

### Evaluation of known common variants associated with NAFLD

Given that four patients did not harbour a rare mutation, yet had lean NAFLD, we evaluated common variants associated with NAFLD and fibrosis (*PNPLA3* rs738409:p.I148M, *GCKR* rs1260326:p.P446L, *TM6SF2* rs58542926:C/T, and MBOAT7-TMC4 rs641738:C/T) and the protective variant *HSD17B13* rs72613567:T/TA. Patient 1, who harboured the rare variant in *ALDOB*, also was heterozygous for the *GCKR* and *MBOAT7* variants, but otherwise wild type for the evaluated single nucleotide polymorphisms. Patient 2, who harboured the rare variant in *APOB*, was homozygous for the risk variant in *PNPLA3* and heterozygous for the variant in *GCKR* and *MBOAT7*. The six lean patients with NAFLD analysed in this cohort were wild type for the risk allele in *TM6SF2* or the protective variants in *HSD17B13* (Table S6). Although patient 6 did not ultimately have a causative variant found on WES analysis, they were found to be homozygous for both the *PNPLA3* and *GCKR* polymorphisms and heterozygous for the *MBOAT7* polymorphisms. Polygenic risk scores incorporating the five variants, calculated as previously described,^13^^,^^14^ varied among the six patients from 0.063 to 0.725.

## Discussion

This study supports the use of WES in the diagnosis and management of lean patients with NAFLD. Two out of 6 patients (33%) with NAFLD without visceral adiposity were discovered to harbour genetic diseases that explain the underlying pathogenesis of their hepatic steatosis. Furthermore, we validated the pathogenicity of the p.Val1856CysfsTer2 variant in *APOB* using MRI quantification of liver fat and *APOB* levels. In the UK Biobank, we found a significant BMI–rare variant interaction on ALT and liver fat, which suggests that adiposity may amplify the effect of rare variants on fatty liver. This finding parallels what has been previously demonstrated for common variants associated with NAFLD.^15^

In a prior study demonstrating the clinical utility of genomic analysis in the diagnosis and management of adults with unexplained liver disease, three out of six non-obese patients with hepatic steatosis in the absence of metabolic syndrome were found to harbour monogenic disorders underlying the triglyceride accumulation seen on hepatocytes.^4^ Subsequently, we have proposed the incorporation of genomic analysis in a variety of liver diseases that remain unexplained despite a comprehensive work-up,^10^^,^^16^ and in a recent review, we proposed a framework for evaluating patients with lean NAFLD. Although patients with lean NAFLD with increased visceral adiposity likely resemble the broader population with NAFLD, those without visceral adiposity may harbour rare monogenic variants that lead to a phenotype mimicking NAFLD. This study applies the proposed framework^5^ to a well-phenotyped cohort of patients with biopsy-proven NAFLD and demonstrates the utility of evaluating patients with lean NAFLD without visceral adiposity for monogenic disorders with a diagnostic yield of 33%. Leveraging the UK Biobank data, we confirmed the pathogenicity of the p.Val1856CysfsTer2 variant in *APOB* and demonstrated an interaction between rare variants in *APOB* and BMI on liver fat and ALT. Heterozygous, rare, predicted loss-of-function variants in *APOB* have been described in other patients with cryptogenic cirrhosis and suggested to contribute to severe disease, including predisposition to hepatocellular carcinoma development.[17], [18], [19], [20], [21]

In this study, the two patients with rare monogenic drivers of disease also had common variants in *PNPLA3* and *GCKR*. Prior studies have demonstrated the need to consider the opposing impact of deleterious and protective variants and demonstrated a similar magnitude of opposing effects of variants in *PNPLA3* and *HSD17B13* on MRE.^22^ When evaluating polygenic risk, consideration of the combination of common and rare variants may refine our understanding of the risk of NAFLD and fibrosis, as has been described in other diseases including cardiovascular disease and breast cancer.^23^

This prospective, systematic assessment of patients with biopsy-proven lean NAFLD using WES adds new information about pathogenic and actionable rare variants in patients with lean NAFLD. Although the sample size is limited, this study involves well-phenotyped patients with detailed information on liver histology and advanced MRI, which differentiates it from large population-based studies in which most rare variant association studies of NAFLD have been performed. Furthermore, the detailed clinical evaluation allowed for confirmation of genotype–phenotype associations. External validation of the clinical significance of the rare variant in *APOB* in the UK Biobank is an additional strength of the study. APOB deficiency should be suspected in patients with NAFLD in the absence of hyperlipidaemia, in whom circulating APOB levels should be examined.

Unveiling the genetic aetiologies of disease in lean patients with NAFLD may lead to more targeted management, genetic screening of family members, and refined disease prognostication, and potentially uncover actionable pathways for drug development. Furthermore, uncovering the heterogeneous molecular drivers of NAFLD and fibrosis may improve future clinical trial design by avoiding enrolment of patients with a subtype of disease unlikely to benefit.^16^ In conclusion, in this well-characterised cohort of patients with biopsy-proven NAFLD, 33% of patients with lean NAFLD without visceral adiposity harboured monogenic disorders associated with fatty liver, highlighting the value of genetic assessment of NAFLD to identify distinct subtypes of disease.

## Financial support

RL receives funding support from 10.13039/100000066NIEHS (5P42ES010337), 10.13039/100006108NCATS (5UL1TR001442), DOD 10.13039/100014042PRCRP (W81XWH-18-2-0026), 10.13039/100000062NIDDK (U01DK061734, R01DK106419, R01DK121378, R01DK124318, and P30DK120515), 10.13039/100000050NHLBI (P01HL147835), and 10.13039/100000027NIAAA (U01AA029019). VA is supported by 10.13039/100000062NIDDK (K23DK119460). SV receives funding from the NIDDK (K08 DK113109 and R01 DK131033) and 10.13039/100000862Doris Duke Charitable Foundation (2019081).

## Conflicts of interest

RL serves as a consultant or advisory board member for Bird Rock Bio, Celgene, Enanta, GRI Bio, Madrigal, Metacrine, NGM, Sanofi, Arrowhead Research, Galmed, NGM, GNI, NovoNordisk, Merck, Siemens, Pfizer, Gilead, and Glympsebio, In addition, his institution has received grant support from Allergan, BMS, BI, Daiichi-Sankyo Inc., Eli-Lilly, Galectin, Galmed, GE, Genfit, Intercept, Janssen Inc, Madrigal, Merck, NGM, Pfizer, Prometheus, Siemens, and Sirius. He is also co-founder of Liponexus Inc. SV serves as a consultant for Albireo Pharma. SA has served as a scientific consultant to Third Rock Ventures. AVK is an employee and holds equity in Verve Therapeutics; has served as a scientific advisor to Amgen, Maze Therapeutics, Navitor Pharmaceuticals, Sarepta Therapeutics, Novartis, Silence Therapeutics, Korro Bio, Veritas International, Color Health, Third Rock Ventures, Illumina, Foresite Labs, and Columbia University (NIH); has received speaking fees from Illumina, MedGenome, Amgen, and the Novartis Institute for Biomedical Research; and has received a sponsored research agreement from IBM Research.

Please refer to the accompanying ICMJE disclosure forms for further details.

## Authors’ contributions

Study concept and design: RL, SV, VA. Data analysis: MZ, DH, CK, SA, SV. Drafting of the manuscript: MZ, SV, VA. Critical revision and approval of the final manuscript: all authors.

## Data availability statement

The datasets generated and/or analysed during the current study are available from the corresponding author on reasonable request in de-identified form.
