# Supplementary material for: Genomic analysis of lean individuals with NAFLD identifies monogenic disorders in a prospective cohort study
Source: JHEP Rep. 2023 Feb 2;5(4):100692. doi: 10.1016/j.jhepr.2023.100692 (PMC10017416; doi:10.1016/j.jhepr.2023.100692)
Supplement: Multimedia component 1 [file mmc1.pdf]

Genomic analysis of lean individuals with NAFLD identifies monogenic disorders in a prospective cohort study

Melanie Zheng, Daniel Huang, Chigoziri Konkwo, Saaket Agrawal, Amit V. Khera, Rohit Loomba, Sílvia Vilarinho, Veeral Ajmera

Table of contents

Supplementary methods..2

Fig. S1..5

Table S1...6

Table S2...7

Table S3...8

Table S4...9

Table S5...10

Table S6...11

Supplementary references..12

Supplementary methods

Inclusion and Exclusion Criteria

Participants meeting any of the following criteria were excluded from the study: significant alcohol consumption (defined as ≥14 drinks/week for men or ≥7 drinks/week for women) within the previous 2-year period; evidence of active substance use. Alcohol intake history was obtained in a clinical setting and verified at the research clinic with the Alcohol Use Disorders Identification Test and the Skinner questionnaire. Other causes of liver disease and hepatic steatosis were ruled out systematically based on history and laboratory tests. Participants were instructed to fast for a minimum of eight hours before collection of laboratory tests.

Whole-exome sequencing and analysis

Germline DNA was extracted from blood samples using standard methods. Germline DNA was captured using IDT xGen exome V2 exome enrichment probes and sequenced using the Illumina NovaSeq platform. Exome sequencing data were mapped and aligned to the reference human genome build 19 using Burrows-Wheeler Aligner.(1) Variants were called using GATK(2) and annotated using Annovar.(3) All variants passed an initial quality control and were filtered out for read depth of coverage < 30 and for segmental duplications (Figure 1). Protein-altering variants were selected by removing synonymous and intronic/non-coding variants. Variants were selected for minor allele frequency (MAF) of <0.01 for homozygous and compound heterozygous variants (recessive inheritance) or $<2 \times 10^{-5}$ for heterozygous variants (dominant inheritance). MAF was determined using the genome aggregation database (gnomAD).(4) Variants were then prioritized based on predicted deleteriousness, using

Combined Annotation Dependent Depletion (CADD)(5) score > 20 for missense variants and SpliceAI(6) score > 0.5 for splice-site variants. Remaining variants were flagged based on an internal list of 264 liver disease-related genes derived from Online Mendelian Inheritance in Man (OMIM) database entries, previously described.(7) Selected NAFLD-associated polymorphisms, namely *PNPLA3* rs738409:p.I148M, *GCKR* rs1260326:p.P446L, *TM6SF2* rs58542926:C/T, and *HSD17B13* rs72613567:T/TA, MBOAT7-TMC4 rs641738:C/T were extracted from WES data.

UK Biobank Cohort and Analysis

Phenotypes

The UK Biobank is an observational study that enrolled over 500,000 individuals between the ages of 40 and 69 years between 2006 and 2010(8). Alanine aminotransferase (UKB field 30620) and apolipoprotein B (UKB field 30640) measured at the time of enrollment were made available to researchers. Imaging-derived liver fat was derived in 36,703 participants of the UK Biobank as previously described(9). This analysis of data from the UK Biobank was approved by the Mass General Brigham institutional review board and was performed under UK Biobank application #7089.

APOB rare variant validation

We conducted rare variant association studies using the first 200,643 exomes from the UK Biobank.(10) An extensive quality control procedure was applied to these data prior to analysis as described elsewhere (11). Following quality control, 200,337 exomes were available for analysis. To identify rare (minor allele frequency < 0.1%)

high-confidence predicted inactivating variants in *APOB*, we applied the previously validated Loss-Of-Function Transcript Effect Estimator (LOFTEE) algorithm implemented within the Ensembl Variant Effect Predictor (VEP) software program as a plugin, VEP version 96.0 (4). We refer to these variants are “LOFHC”.

Statistical analysis

All effect sizes are reported from linear regressions adjusted for age (at enrollment for ALT and APOB, at the time of imaging for liver fat %), sex, and the first 10 principal components of genetic ancestry (liver fat analyses were additionally adjusted for MRI serial number). Prior to analysis, one sample of a pair was randomly excluded if that pair had second-degree relative or closer kinship. Carrier counts in Figure 2 are reported following this exclusion and correspond to participants with the studied phenotype available. Analyses were repeated in low (< 25 kg/m^2) and high (>= 25 kg/m^2) body mass index (BMI) subgroups. The interaction between *APOB* rare variant carrier status and BMI was tested using a linear regression including BMI and a carrier status by BMI interaction term along with the above covariates. All analyses were performed using R 3.6.0.

Fig. S1. Whole exome sequencing variant filtering pipeline revealing the pathogenic mutation in *ALDOB* for Patient 1 and *APOB* for Patient 2. MAF, minor allele frequency; CADD, Combined Annotation Dependent Depletion; OMIM, Online Mendelian Inheritance in Man.
*list from Zheng M, Allington G, Vilarinho S. Genomic medicine for liver disease. *Hepatology.* 2022.

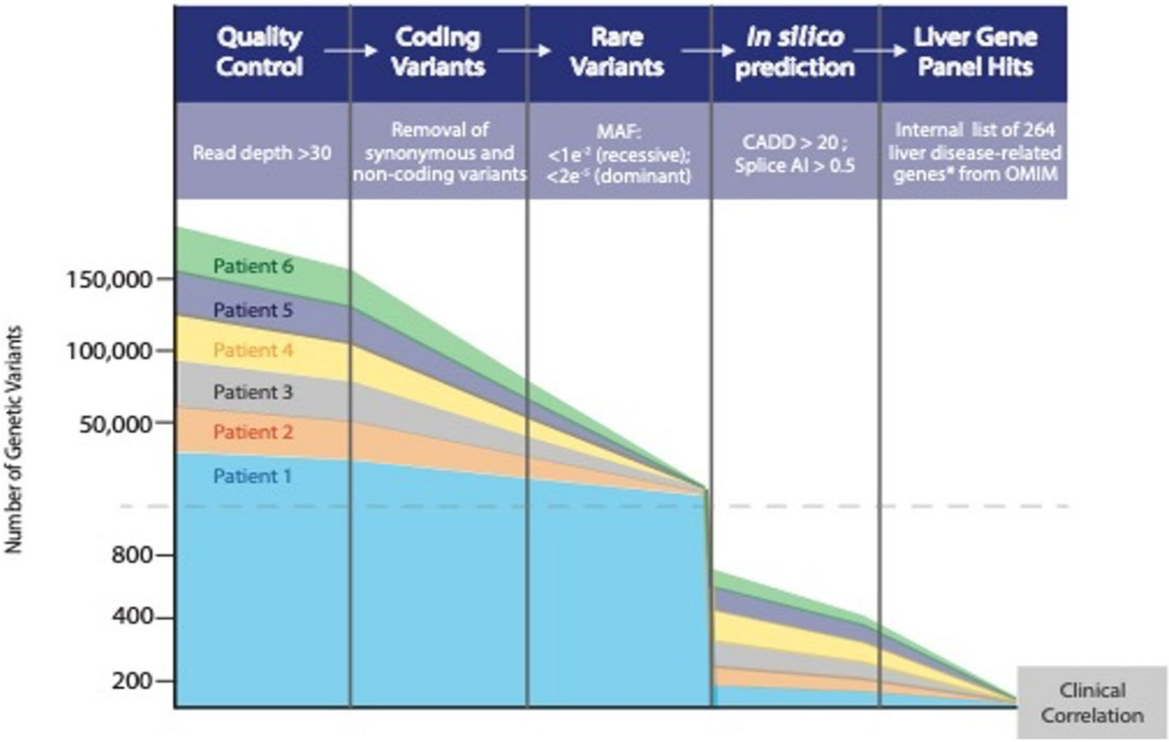

Table S1. Baseline characteristics of participants with NAFLD, stratified by baseline body mass index

	**Overall** (N=124)	**Non-lean** (N=118)	**Lean** (N=6)	**P value**
**Demographic profile**				
Age (yr)	57.00 [47.75, 65.00]	57.00 [47.25, 65.00]	59.50 [53.75, 63.00]	0.834
Female, n (%)	78 (62.9)	73 (61.9)	5 (83.3)	0.529
BMI (kg/m^2)	31.82 [29.00, 36.53]	32.24 [29.40, 36.90]	23.42 [21.74, 24.54]	<0.001
Diabetes Mellitus, n (%)	61 (49.2)	58 (49.2)	3 (50.0)	1
Hispanic, n (%)	48 (38.7)	47 (39.8)	1 (16.7)	0.48
**Biochemical data**				
AST (u/L)	35.00 [26.00, 59.00]	35.00 [26.00, 58.75]	41.50 [36.50, 72.75]	0.292
ALT (u/L)	46.50 [32.00, 78.00]	46.50 [32.00, 77.50]	45.50 [43.25, 120.50]	0.386
HbA1c (%)	6.00 [5.60, 6.70]	6.00 [5.60, 6.70]	5.95 [5.67, 6.15]	0.834
Total Bilirubin (mg/dl)	0.49 [0.30, 0.60]	0.49 [0.30, 0.60]	0.50 [0.35, 0.65]	0.787
Direct Bilirubin (mg/dl)	0.10 [0.10, 0.19]	0.10 [0.10, 0.19]	0.15 [0.10, 0.20]	0.963
INR	1.00 [1.00, 1.10]	1.00 [1.00, 1.10]	1.05 [1.00, 1.10]	0.516
Albumin (g/dl)	4.40 [4.30, 4.60]	4.40 [4.30, 4.60]	4.70 [4.55, 4.77]	0.027
Total cholesterol (mg/dL)	182.50 [156.75, 207.50]	182.50 [156.25, 206.75]	188.00 [167.00, 215.00]	0.692
HDL (mg/dL)	43.00 [36.00, 54.25]	43.00 [36.00, 54.00]	46.00 [40.50, 56.75]	0.552
LDL (mg/dL)	100.50 [82.75, 124.50]	100.50 [82.25, 125.50]	110.00 [90.25, 120.75]	0.847
TG (mg/dL)	142.00 [110.00, 196.25]	142.00 [110.25, 196.00]	149.50 [103.50, 191.00]	0.912
Interval between biopsies (months)	17.85 [8.47, 32.62]	17.85 [8.43, 33.47]	17.35 [13.53, 24.18]	0.87
**Liver histology findings baseline**				
NAS	5.00 [4.00, 6.00]	5.00 [4.00, 6.00]	5.50 [4.25, 6.00]	0.7
Fibrosis stage, n (%)				
0	34 (27.4)	33 (28.0)	1 (16.7)	0.59
1	45 (36.3)	44 (37.3)	1 (16.7)	
2	15 (12.1)	14 (11.9)	1 (16.7)	
3	19 (15.3)	17 (14.4)	2 (33.3)	
4	11 (8.9)	10 (8.5)	1 (16.7)	
Steatosis score, n (%)				
0	1 (0.8)	1 (0.8)	0 (0.0)	0.838
1	41 (33.1)	40 (33.9)	1 (16.7)	
2	49 (39.5)	46 (39.0)	3 (50.0)	
3	33 (26.6)	31 (26.3)	2 (33.3)	
Lobular inflammation score, n (%)				
0	0	0	0	0.793
1	45 (36.6)	43 (36.8)	2 (33.3)	
2	71 (57.7)	67 (57.3)	4 (66.7)	
3	7 (5.7)	7 (6.0)	0 (0.0)	
Ballooning score, n (%)				
0	12 (9.7)	11 (9.3)	1 (16.7)	0.833
1	70 (56.5)	67 (56.8)	3 (50.0)	
2	42 (33.9)	40 (33.9)	2 (33.3)	
**Imaging results**				
MRI-PDFF (%)	14.05 [8.69, 19.50]	13.90 [8.62, 19.35]	18.89 [14.57, 23.40]	0.179
MR elastography	2.92 [2.50, 3.89]	2.92 [2.50, 3.88]	3.03 [2.82, 3.70]	0.936

Median values are provided with IQR in parenthesis, unless otherwise noted as n (%)
Abbreviations: NAS, NAFLD activity score; yr, year; SD, standard deviation; AST, aspartate transaminase; ALT, alanine transaminase; HbA1c, hemoglobin A1c; MRI-PDFF, magnetic resonance imaging – proton density fat fraction
Categorical variables tested with chi square.
Continuous variables compared using t-test or Wilcoxon two-sample test as appropriate.

Table S2. Change in fibrosis stage in participants with NAFLD, stratified by body mass index

	**Non-lean** (N=118)	**Lean** (N=6)	p-value
**Change in fibrosis stage**			
-2	2 (1.7)	0 (0.0)	0.413
-1	23 (19.5)	0 (0.0)	
0	63 (53.4)	6 (100.0)	
1	25 (21.2)	0 (0.0)	
2	4 (3.4)	0 (0.0)	
3	1 (0.8)	0 (0.0)	
**Fibrosis progression**	30 (25.4)	0 (0.0)	0.352
**Fibrosis regression**	25 (21.2)	0 (0.0)	0.459
**Change in NAS**	-1.00 [-2.00, 0.00]	-1.50 [-2.00, -1.00]	0.495
**Change in MRE (kPa)**	-0.14 [-0.54, 0.32]	-0.12 [-0.16, -0.12]	0.707
**Change in MRI-PDFF**	-1.17 [-4.03, 1.70]	-4.00 [-4.10, -1.75]	0.521

Values reported as n (%)

Table S3: Whole-exome sequencing metrics of the lean patient cohort with NAFLD (n=6).

Variable	Mean	Range
Mean depth (x)	67.1	(63.4 - 70.1)
% Error rate	0.358	(0.34 - 0.38)
% of targeted bases >8 independent reads	98	(98.0 - 98.0)
% of targeted bases >30 independent reads	94.8	(94.1 - 95.5)

Table S4: Table 2. Summary of genotype-phenotype data for the two adult lean patients with type 2 NAFLD who were found to have monogenic diagnoses.

**Genotype Information**									
**Patient ID**	Affected Gene	Zygosity	AA Mutation/ Consequence	gnomAD MAF (overall)	gnomAD MAF (max sub-population)	CADD score	MetaSVM score	**Clinical Phenotype**	**Genetic Diagnosis**
1	*ALDOB*	Homozygous	p.Ala150Pro	$3.093e^{-3}$	$4.866e^{-3}$ (non-Finnish European)	31	0.511	NAFLD type 2	Hereditary fructose intolerance
2	*APOB*	Heterozygous	p.Val1856CysfsTer2	$3.979e^{-6}$	$6.153e^{-5}$ (African)	N/A	N/A		Familial hypobetalipoproteinemia

AA, amino acid; gnomAD, Genome Aggregation Database; MAF, minor allele frequency; CADD, Combined Annotation Dependent Depletion; MetaSVM, Meta-analytic support vector machine; N/A, not applicable

Table S5: Enrichment of LOFHC variants in *APOB*, *MTTP*, and *TM6SF2* in UK Biobank participants with low apolipoprotein B

	Genotype Information				
Trait	Variant Set	Case Frequency (%)	Control Frequency (%)	Odds Ratio (95% CI)	P-value
Bottom 10% apolipoprotein B (apoB <= 0.74 g/L)	*APOB* LOFHC	130/17,424 (0.75%)	42/157,917 (0.03%)	29.2 (20.6 – 41.4)	6.0×10^{-95}
	MTTP LOFHC	24/17,424 (0.14%)	105/157,917 (0.07%)	2.1 (1.4 – 3.3)	1.2×10^{-3}
	TM6SF2 LOFHC	10/17,424 (0.06%)	56/157,917 (0.04%)	1.7 (0.9 – 3.3)	0.16

Participants were dichotomized by whether they were in the bottom decile of apolipoprotein B in the UK Biobank. Among the 200,337 UK Biobank exomes following quality control, 294 participants harbored one of 105 LOFTEE-derived high confidence predicted loss-of function (LOFHC) variants in *APOB* (including the p.Val1856CysfsTer2 variant discussed in the main text), 145 participants harbored one of 29 LOFHC variants in *MTTP*, and 75 participants harbored one of 17 LOFHC variants in *TM6SF2*. Following quality control and removal of related samples (see Methods), 175,341 UK Biobank participants were available for the present analysis. Effect sizes and standard errors used to generate 95% confidence intervals were obtained from Firth logistic regression, while p-values were obtained from the SPA test, both as implemented in the R package SPAtest. Models were adjusted for age, sex, and the first ten principal components of genetic ancestry. Note that the number of carriers listed in the table is fewer than those reported in the legend because of (1) removal of related samples prior to analysis and (2) several rare variant carriers having missing apolipoprotein B.

Table S6: Evaluation of common variants and polygenic risk score associated with NAFLD and fibrosis in lean NAFLD patients

	Genotype Information					
Patient ID	PNPLA3 rs738409:p.I148M	GCKR rs1260326:p.P446L	TM6SF2 rs58542926:C/T	HSD17B13 rs72613567:T/TA	MBOAT7-TMC4 rs641738:C/T	Polygenic Risk Score*
1	Wild-type	Heterozygous	Wild-type	Wild-type	Heterozygous	0.128
2	Homozygous	Heterozygous	Wild-type	Wild-type	Heterozygous	0.66
3	Wild-type	Wild-type	Wild-type	Wild-type	Heterozygous	0.063
4	Heterozygous	Heterozygous	Wild-type	Wild-type	Heterozygous	0.394
5	Heterozygous	Wild-type	Wild-type	Wild-type	Homozygous	0.392
6	Homozygous	Homozygous	Wild-type	Wild-type	Heterozygous	0.725

*Polygenic risk score calculated as previously described, 0.266*PNPLA3 + 0.274*TM6SF2 + 0.065*GCKR + 0.063*MBOAT7 – 0.361*HSD17B13 (12, 13)

Supplementary references

1. Li H, Durbin R. Fast and accurate short read alignment with Burrows-Wheeler transform. Bioinformatics 2009;25:1754-1760.
2. Van der Auwera GA, Carneiro MO, Hartl C, Poplin R, del Angel G, Levy-Moonshine A, Jordan T, et al. From FastQ Data to High-Confidence Variant Calls: The Genome Analysis Toolkit Best Practices Pipeline. Current Protocols in Bioinformatics 2013;43:11.10.11-11.10.33.
3. Wang K, Li M, Hakonarson H. ANNOVAR: functional annotation of genetic variants from high-throughput sequencing data. Nucleic Acids Research 2010;38:e164-e164.
4. Karczewski KJ, Francioli LC, Tiao G, Cummings BB, Alföldi J, Wang Q, Collins RL, et al. The mutational constraint spectrum quantified from variation in 141,456 humans. Nature 2020;581:434-443.
5. Rentzsch P, Witten D, Cooper GM, Shendure J, Kircher M. CADD: predicting the deleteriousness of variants throughout the human genome. Nucleic Acids Research 2019;47:D886-D894.
6. Jaganathan K, Kyriazopoulou Panagiotopoulou S, McRae JF, Darbandi SF, Knowles D, Li YI, Kosmicki JA, et al. Predicting Splicing from Primary Sequence with Deep Learning. Cell 2019;176:535-548.e524.
7. Zheng M, Allington G, Vilarinho S. Genomic medicine for liver disease. Hepatology 2022;76:860-868.
8. Sudlow C, Gallacher J, Allen N, Beral V, Burton P, Danesh J, Downey P, et al. UK biobank: an open access resource for identifying the causes of a wide range of complex diseases of middle and old age. PLoS Med 2015;12:e1001779.
9. Haas ME, Pirruccello JP, Friedman SN, Wang M, Emdin CA, Ajmera VH, Simon TG, et al. Machine learning enables new insights into genetic contributions to liver fat accumulation. Cell Genom 2021;1.
10. Szustakowski JD, Balasubramanian S, Kvikstad E, Khalid S, Bronson PG, Sasson A, Wong E, et al. Advancing human genetics research and drug discovery through exome sequencing of the UK Biobank. Nat Genet 2021;53:942-948.
11. Jurgens SJ, Choi SH, Morrill VN, Chaffin M, Pirruccello JP, Halford JL, Weng LC, et al. Analysis of rare genetic variation underlying cardiometabolic diseases and traits among 200,000 individuals in the UK Biobank. Nat Genet 2022;54:240-250.
12. Dongiovanni P, Stender S, Pietrelli A, Mancina RM, Cespiati A, Petta S, Pelusi S, et al. Causal relationship of hepatic fat with liver damage and insulin resistance in nonalcoholic fatty liver. J Intern Med 2018;283:356-370.
13. Bianco C, Jamialahmadi O, Pelusi S, Baselli G, Dongiovanni P, Zanoni I, Santoro L, et al. Non-invasive stratification of hepatocellular carcinoma risk in non-alcoholic fatty liver using polygenic risk scores. J Hepatol 2021;74:775-782.
