# Supplementary material for: Genomic analysis of lean individuals with NAFLD identifies monogenic disorders in a prospective cohort study
Source: JHEP Rep. 2023 Feb 2;5(4):100692. doi: 10.1016/j.jhepr.2023.100692 (PMC10017416; doi:10.1016/j.jhepr.2023.100692)
Supplement: Multimedia component 2 [file mmc2.docx]

**Journal of Hepatology**

**CTAT methods**

Tables for a “Complete, Transparent, Accurate and Timely account” (CTAT) are now mandatory for all revised submissions. The aim is to enhance the reproducibility of methods.

- Only include the parts relevant to your study
- Refer to the CTAT in the main text as ‘Supplementary CTAT Table’
- Do not add subheadings
- Add as many rows as needed to include all information
- Only include one item per row

**If the CTAT form is not relevant to your study, please outline the reasons why:**

|  |
| --- |

- 1. **Antibodies**

| **Name** | **Citation** | **Supplier** | **Cat no.** | **Clone no.** |
| --- | --- | --- | --- | --- |
|  |  |  |  |  |

- 1. **Cell lines**

| **Name** | **Citation** | **Supplier** | **Cat no.** | **Passage no.** | **Authentication test method** |
| --- | --- | --- | --- | --- | --- |
|  |  |  |  |  |  |

- 1. **Organisms**

| **Name** | **Citation** | **Supplier** | **Strain** | **Sex** | **Age** | **Overall n number** |
| --- | --- | --- | --- | --- | --- | --- |
|  |  |  |  |  |  |  |

- 1. **Sequence based reagents**

| **Name** | **Sequence** | **Supplier** |
| --- | --- | --- |
|  |  |  |

- 1. **Biological samples**

| **Description** | **Source** | **Identifier** |
| --- | --- | --- |
| Blood samples | University of California San Diego NAFLD Research Center | Biopsy-proven NAFLD Cohort |

- 1. **Deposited data**

| **Name of repository** | **Identifier** | **Link** |
| --- | --- | --- |
|  |  |  |

- 1. **Software**

| **Software name** | **Manufacturer** | **Version** |
| --- | --- | --- |
| GATK | Broad Institute | 4.3.0.0 |
| Annovar | Annovar | 2019Oct24 |
| CADD (Combined Annotation Dependent Depletion) | University of Washington, Hudson-Alpha Institute for Biotechnology and Berlin Institute of Health | v1.6 |
| SpliceAI | Broad Institute | v37 |

- 1. **Other (e.g. drugs, proteins, vectors etc.)**

|  |  |  |
| --- | --- | --- |
|  |  |  |

- 1. **Please provide the details of the corresponding methods author for the manuscript:**

| Equal contribution  Veeral Ajmera, MD, MAS  Division of Gastroenterology and Hepatology  University of California, San Diego  1W507, ACTRI Building  La Jolla, CA, 92093-0887, USA  Email: v1ajmera@ucsd.edu  or  Sílvia Vilarinho, MD, PhD  Departments of Internal Medicine (Digestive Diseases) and of Pathology,  Yale School of Medicine  300 Cedar Street  TAC building, S-231  New Haven, CT 06510  Email: [silvia.vilarinho@yale.edu](mailto:silvia.vilarinho@yale.edu)  or  Rohit Loomba, MD, MHSc  Director, NAFLD Research Center  Division of Gastroenterology and Hepatology  1W202, ACTRI Building  La Jolla, CA, 92093-0887, USA  Email: [roloomba@ucsd.edu](mailto:roloomba@ucsd.edu) |
| --- |

**2.0 Please confirm for randomised controlled trials all versions of the clinical protocol are included in the submission. These will be published online as supplementary information.**

|  |
| --- |
