# Supplementary material for: Genomic analysis of lean individuals with NAFLD identifies monogenic disorders in a prospective cohort study
Source: JHEP Rep. 2023 Feb 2;5(4):100692. doi: 10.1016/j.jhepr.2023.100692 (PMC10017416; doi:10.1016/j.jhepr.2023.100692)
Supplement: Multimedia component 4 [file mmc4.pdf]

Genomic analysis of lean individuals with NAFLD identifies monogenic disorders in a prospective cohort study

Melanie Zheng,[1] Daniel Q. Huang,[2,3] Chigoziri Konkwo,[1] Saaket Agrawal,[4] Amit V. Khera,[4,5] Rohit Loomba,[2,*,†] Sílvia Vilarinho,[1,6,*,†] Veeral Ajmera[2,*,†]

[1]Departments of Internal Medicine, Section of Digestive Diseases, and of Pathology, Yale School of Medicine, New Haven, CT, USA; [2]NAFLD Research Center, Division of Gastroenterology, University of California at San Diego, La Jolla, CA, USA; [3]Department of Medicine, Yong Loo Lin School of Medicine, National University of Singapore, Singapore; [4]Program in Medical and Population Genetics, Broad Institute of MIT and Harvard, Cambridge, MA, USA; [5]Verve Therapeutics, Cambridge, MA, USA; [6]Department of Pathology, Yale School of Medicine, New Haven, CT, USA

JHEP Reports **2023.** https://doi.org/10.1016/j.jhepr.2023.100692

Background & Aims: Lean patients with non-alcoholic fatty liver disease (NAFLD) represent 10–20% of the affected population and may have heterogeneous drivers of disease. We have recently proposed the evaluation of patients with lean NAFLD without visceral adiposity for rare monogenic drivers of disease. Here, we aimed to validate this framework in a well-characterised cohort of patients with biopsy-proven NAFLD by performing whole exome sequencing.
Methods: This prospective study included 124 patients with biopsy-proven NAFLD and paired liver biopsies who underwent standardised research visits including advanced magnetic resonance imaging (MRI) assessment of liver fat and stiffness.
Results: Six patients with lean NAFLD were identified and underwent whole exome sequencing. Two lean patients (33%) were identified to have monogenic disorders. The lean patients with monogenic disorders had similar age, and anthropometric and MRI characteristics to lean patients without a monogenic disorder. Patient 1 harbours a rare homozygous pathogenic mutation in *ALDOB* (aldolase B) and was diagnosed with hereditary fructose intolerance. Patient 2 harbours a rare heterozygous mutation in apolipoprotein B (*APOB*). The pathogenicity of this *APOB* variant (p.Val1856CysfsTer2) was further validated in the UK Biobank and associated with lower circulating APOB levels (beta = -0.51 g/L, 95% CI -0.65 to -0.36 g/L, $p = 1.4 \times 10^{-11}$) and higher liver fat on MRI (beta = +10.4%, 95% CI 4.3–16.5%, $p = 8.8 \times 10^{-4}$). Hence, patient 2 was diagnosed with heterozygous familial hypobetalipoproteinaemia.
Conclusions: In this cohort of well-characterised patients with lean NAFLD without visceral adiposity, 33% (2/6) had rare monogenic drivers of disease, highlighting the importance of genomic analysis in this NAFLD subtype.
Impact and Implications: Although most people with non-alcoholic fatty liver disease (NAFLD) are overweight or obese, a subset are lean and may have unique genetic mutations that cause their fatty liver disease. We show that 33% of study participants with NAFLD who were lean harboured unique mutations that cause their fatty liver, and that these mutations had effects beyond the liver. This study demonstrates the value of genetic assessment of NAFLD in lean individuals to identify distinct subtypes of disease.
© 2023 The Author(s). Published by Elsevier B.V. on behalf of European Association for the Study of the Liver (EASL). This is an open access article under the CC BY license (http://creativecommons.org/licenses/by/4.0/).

Introduction

The prevalence of non-alcoholic fatty liver disease (NAFLD) continues to grow, affecting an estimated 60–80 million people in the United States, and a subset of patients will progress to advanced liver disease including cirrhosis and hepatocellular carcinoma.[1] Patients with NAFLD who are lean represent 10–20% of the total population, and observational studies have yielded conflicting results with regard to disease severity and prognosis,[2,3] which may be related to more heterogeneous drivers of disease.

A previous study demonstrating the clinical utility of genomic analysis in the diagnosis and management of adult patients with liver disease of unknown aetiology revealed previously unappreciated monogenic disorders in three non-obese patients with NAFLD.[4] This finding led us to propose a framework for genomic evaluation of lean patients with NAFLD who lack visceral adiposity, to identify rare genetic variants that may have therapeutic implications and elucidate additional pathogenic mechanisms.[5] Here, we performed whole exome sequencing (WES) on lean individuals from a well-phenotyped longitudinal cohort with biopsy-proven NAFLD to evaluate for rare, monogenic drivers of disease.

Keywords: NAFLD; Non-obese; Rare genetic variants; Whole exome sequencing.
Received 29 November 2022; received in revised form 13 January 2023; accepted 17 January 2023; available online 2 February 2023
† These authors contributed equally.
* Corresponding authors. Addresses: Division of Gastroenterology and Hepatology, University of California, San Diego, 1W507, ACTRI Building, La Jolla, CA, 92093-0887, USA (V. Ajmera); Departments of Internal Medicine (Digestive Diseases) and of Pathology, Yale School of Medicine, 300 Cedar Street, TAC building, S-231, New Haven, CT, 06510, USA (S. Vilarinho); NAFLD Research Center, Division of Gastroenterology and Hepatology, 1W202, ACTRI Building, La Jolla, CA, 92093-0887, USA (R. Loomba).
E-mail addresses: v1ajmera@ucsd.edu (V. Ajmera), silvia.vilarinho@yale.edu (S. - Vilarinho), roloomba@ucsd.edu (R. Loomba). .

ELSEVIER

EASL The Home of Hepatology

Patients and methods

This is a longitudinal study derived from a well-characterised prospective cohort of patients with biopsy-proven NAFLD and paired liver biopsies who underwent a standard research visit that included history, physical examination, biochemical testing, and paired liver biopsy assessment (using the Non-alcoholic Steatohepatitis Clinical Research Network histologic scoring system) at the University of California San Diego (UCSD) NAFLD Research Center from 2006 through 2019. All patients provided written informed consent before enrolling in the study, and the study was approved by the UCSD Institutional Review Board. At baseline, all patients underwent a standardised clinical evaluation including detailed history, anthropometric exam, and laboratory testing at the UCSD NAFLD Research Center. Patients ≥18 years of age with biopsy-proven NAFLD were included and were identified as lean NAFLD by BMI ≤ 25 kg/m^2 for non-Asians and ≤ 23 kg/m^2 for Asians. Germline DNA was extracted from blood samples using standard methods. Germline DNA was captured using xGen exome V2 exome enrichment probes (Integrated DNA Technologies Coralville, Iowa) and sequenced using the Illumina NovaSeq platform (San Diego, California). The apolipoprotein B (*APOB*) rare variant validation was performed in the UK Biobank. All analyses were performed using R 3.6.0 (R Foundation for Statistical Computing, Vienna, Austria). Additional details are provided in the Supplementary information.

Results

Of 124 participants with biopsy-proven NAFLD who had longitudinal follow-up,[6] six patients with lean NAFLD, defined as a BMI ≤ 25 kg/m^2 for non-Asians and ≤ 23 kg/m^2 for Asians, were identified. Lean and non-lean participants were similar with regard to age, sex, diabetes status, laboratory parameters, liver histology, and magnetic resonance imaging proton density fat fraction (MRI-PDFF) and magnetic resonance elastography (MRE) (Table S1). There was no difference in longitudinal change in histology, MRI-PDFF, or MRE between patients with lean NAFLD and those with non-lean NAFLD (Table S2). Six participants with lean NAFLD underwent whole exome sequencing (WES) of germline DNA (Table S3). Using the WES analysis pipeline (Fig. S1), we identified a monogenic disorder in two of these adult lean individuals with NAFLD (Table S4). Lean patients with monogenic disorders were of similar age and BMI and had similar fasting insulin levels to lean patients without an identified monogenic disorder (Table 1). None of the patients in this study with biopsy-proven lean NAFLD harboured the protective rare variant in cell death-inducing DFFA-like effector B.

Diagnosis of hereditary fructose intolerance in patient 1

Patient 1 had a BMI of 21.3 kg/m^2, with biopsy-proven non-alcoholic steatohepatitis with stage 2 fibrosis and MRI-PDFF of 24% consistent with severe steatosis. She was found to harbour a rare homozygous missense variant (chr9:104189856; C>G; p.Ala150Pro) in *ALDOB*, which encodes aldolase B. Biallelic variants in this gene cause hereditary fructose intolerance. Aldolase B is the enzyme responsible for catalysing fructose 1,6-bisphosphate into glyceraldehyde 3-phosphate and dihydroxyacetone phosphate, and of fructose 1-phosphate into glyceraldehyde and dihydroxyacetone phosphate. Given the toxic metabolite accumulation as a result of the ingestion of fructose or sucrose, affected patients may present with hypoglycaemia, hepatic steatosis, and proximal renal tubulopathy.[7] This variant was predicted to be damaging by the additional *in silico* prediction models MetaSVM, SIFT, and PolyPhen-2 and has been reported as pathogenic in the ClinVar National Center for Biotechnology Information database. Experimental studies have shown that this missense mutation reduces substrate affinity and enzyme stability and activity within aldolase B.[8] This variant in homozygosity or compound heterozygosity has been described in individuals affected with hereditary fructose intolerance. This patient reported nausea, abdominal pain, and hypoglycaemia exacerbated by fruit intake consistent with hereditary fructose intolerance. The patient had no family history of hereditary fructose intolerance in her first-degree relatives.

Diagnosis of FHBL in patient 2

Patient 2 had a BMI of 24.96 kg/m^2, with biopsy-proven non-alcoholic steatohepatitis with stage 3 fibrosis and MRI-PDFF of 14%. WES of germline DNA from patient 2 revealed a heterozygous frameshift variant (chr2:21234172, AAC>A; p.Val1856CysfsTer2) in *APOB*. APOB is the primary apolipoprotein of chylomicrons and VLDL, IDL, and LDL particles.[9] Familial hypobetalipoproteinaemia (FHBL) presents with low circulating lipid levels and increased hepatic steatosis.[4,5] This frameshift variant has been reported as pathogenic in the ClinVar National Center for Biotechnology Information (NCBI) database and previously associated with FHBL, but it has also been annotated as likely benign in the ClinVar NCBI database. Hence, we went back to the patient to perform genotype–phenotype correlation.[4,10] In addition to hepatic steatosis, the patient had low circulating lipid levels (LDL = 47 mg/dl, total cholesterol = 102 mg/dl, and triglycerides = 66 mg/dl) The patient's APOB level was evaluated and was low at 39 mg/dl. She had no family history of hypobetalipoproteinaemia in first-degree relatives. We next studied the first 200,643 exome-sequenced participants of the UK Biobank to better characterise the clinical significance of the p.Val1856CysfsTer2 variant in *APOB*.[11] Following a previously described genetic and sample quality control pipeline,[12] we identified 14 (0.007%) heterozygous carriers of p.Val1856CysfsTer2, two of whom returned for a follow-up imaging visit for MRI-derived liver fat measurement. Carriers of p.Val1856CysfsTer2 had lower APOB levels (beta = -0.51 g/L, 95% CI -0.65 to -0.36 g/L, p = 1.4 × 10^{-11}), higher liver fat (beta = +10.4%, 95% CI 4.3–16.5%, p = 8.8 × 10^{-4}), and a trend toward higher alanine aminotransferase (ALT) (beta =+6.7 U/L, 95% CI -0.4 to 13.8 U/L, p = 0.07). We next studied participants who carried loss-of-function transcript effect estimator (LOFTEE)-derived high-confidence predicted loss-of-function (LOFHC) variants in *APOB*, excluding p.Val1856CysfsTer2. We observed 280 heterozygote carriers of LOFHC variants in *APOB* (21 with liver imaging) across 104 variants, all with minor allele frequency less than 0.01%. Associations with ALT, APOB, and liver fat in these carriers were comparable with those of the p.Val1856CysfsTer2 variant (Fig. 1). In addition, UK Biobank participants with low serum APOB levels were more likely to harbour LOFHC variants in *APOB* and microsomal triglyceride transfer protein (MTTP) (Table S5). Finally, we evaluated the interaction between BMI and *APOB* variants combining p.Val1856CysfsTer2 with all other LOFHC variants in *APOB* and found a positive interaction term for both ALT (p = 0.008) and liver fat (p = 6.3 × 10^{-5}), suggesting that higher BMI amplifies the impact of the studied rare *APOB* variants on pathologic liver traits. Altogether, genotype and

Table 1. Baseline characteristics of participants with lean NAFLD, stratified by presence of pathogenic mutations.

	All lean patients (n = 6)	No monogenic mutations (n = 4)	Monogenic mutations (n = 2)	*p* value
**Demographic profile**				
Age (years)	59.50 [53.75, 63.00]	59.50 [51.25, 63.00]	61.00 [57.00, 65.00]	0.639
Female, n (%)	5 (83.3)	3 (75.0)	2 (100.0)	1
BMI (kg/m^2)	23.42 [21.74, 24.54]	23.42 [22.43, 24.23]	23.13 [22.22, 24.04]	1
Diabetes mellitus, n (%)	3 (50.0)	3 (75.0)	0 (0.0)	0.4
Hispanic, n (%)	1 (16.7)	1 (25.0)	0 (0.0)	1
**Biochemical data**				
AST (U/L)	41.50 [36.50, 72.75]	38.00 [32.75, 59.00]	62.50 [52.25, 72.75]	0.355
ALT (U/L)	45.50 [43.25, 120.50]	45.00 [41.75, 91.50]	94.50 [69.25, 119.75]	0.643
HbA1c (%)	5.95 [5.67, 6.15]	5.80 [5.60, 6.05]	6.45 [6.18, 6.72]	0.348
Total bilirubin (mg/dl)	0.50 [0.35, 0.65]	0.50 [0.30, 0.70]	0.50 [0.50, 0.50]	1
Direct bilirubin (mg/dl)	0.15 [0.10, 0.20]	0.15 [0.10, 0.19]	0.15 [0.12, 0.17]	0.812
INR	1.05 [1.00, 1.10]	1.10 [1.08, 1.10]	1.00 [1.00, 1.00]	0.114
Albumin (g/dl)	4.70 [4.55, 4.77]	4.60 [4.47, 4.78]	4.75 [4.73, 4.77]	0.481
Total cholesterol (mg/dl)	188.00 [167.00, 215.00]	188.00 [171.00, 212.50]	160.50 [131.25, 189.75]	0.643
HDL (mg/dl)	46.00 [40.50, 56.75]	45.00 [38.25, 52.25]	57.00 [49.50, 64.50]	0.355
LDL (mg/dl)	110.00 [90.25, 120.75]	110.00 [96.75, 127.75]	84.00 [65.50, 102.50]	0.643
TG (mg/dl)	149.50 [103.50, 191.00]	183.00 [148.75, 211.50]	99.00 [82.50, 115.50]	0.165
Insulin	13.00 [8.00, 23.00]	13.00 [10.50, 25.50]	14.50 [10.25, 18.75]	0.564
Interval between biopsies (months)	17.35 [13.53, 24.18]	13.65 [11.48, 15.62]	30.75 [28.03, 33.48]	0.064
**Liver histology findings baseline**				
NAS	5.50 [4.25, 6.00]	4.50 [3.75, 5.25]	6.00 [6.00, 6.00]	0.14
Fibrosis stage, n (%)				1
0	1 (16.7)	1 (25.0)	0 (0.0)	
1	1 (16.7)	1 (25.0)	0 (0.0)	
2	1 (16.7)	0 (0.0)	1 (50.0)	
3	2 (33.3)	1 (25.0)	1 (50.0)	
4	1 (16.7)	1 (25.0)	0 (0.0)	
Steatosis score, n (%)				0.467
0	0 (0.0)	0	0	
1	1 (16.7)	2 (50.0)	0 (0.0)	
2	3 (50.0)	2 (50.0)	2 (100.0)	
3	2 (33.3)	2 (50.0)	0 (0.0)	
Lobular inflammation score, n (%)				0.467
0	0	0	0	
1	2 (33.3)	2 (50.0)	0 (0.0)	
2	4 (66.7)	2 (50.0)	2 (100.0)	
3	0 (0.0)	0	0	
Ballooning score, n (%)				1
0	1 (16.7)	1 (25.0)	0 (0.0)	
1	3 (50.0)	2 (50.0)	1 (50.0)	
2	2 (33.3)	1 (25.0)	1 (50.0)	
**Imaging results**				
MRI-PDFF (%)	18.89 [14.57, 23.40]	18.89 [14.08, 23.46]	19.02 [16.51, 21.53]	1
MRE	3.03 [2.82, 3.70]	3.03 [2.69, 3.40]	3.34 [3.06, 3.61]	1

Median values are provided with IQR in brackets, unless otherwise noted as n (%). Categorical variables tested using the Fisher exact test. Continuous variables compared using the *t* test or Wilcoxon's two-sample test, as appropriate. ALT, alanine transaminase; AST, aspartate transaminase; HbA_{1c}, haemoglobin A_{1c}; INR, international normalised ratio; MRE, magnetic resonance elastography; MRI-PDFF, magnetic resonance imaging proton density fat fraction; NAS, NAFLD activity score; TG, triglycerides.

phenotype findings, as well as external validation of the impact of this rare variant, support that this patient has autosomal dominant *APOB*-related FHBL.

Evaluation of known common variants associated with NAFLD

Given that four patients did not harbour a rare mutation, yet had lean NAFLD, we evaluated common variants associated with NAFLD and fibrosis (*PNPLA3* rs738409:p.I148M, *GCKR* rs1260326:p.P446L, *TM6SF2* rs58542926:C/T, and MBOAT7-TMC4 rs641738:C/T) and the protective variant *HSD17B13* rs72613567:T/TA. Patient 1, who harboured the rare variant in *ALDOB*, also was heterozygous for the *GCKR* and *MBOAT7* variants, but otherwise wild type for the evaluated single nucleotide polymorphisms. Patient 2, who harboured the rare variant in *APOB*, was homozygous for the risk variant in *PNPLA3* and heterozygous for the variant in *GCKR* and *MBOAT7*. The six lean patients with NAFLD analysed in this cohort were wild type for the risk allele in *TM6SF2* or the protective variants in *HSD17B13* (Table S6). Although patient 6 did not ultimately have a causative variant found on WES analysis, they were found to be homozygous for both the *PNPLA3* and *GCKR* polymorphisms and heterozygous for the *MBOAT7* polymorphisms. Polygenic risk scores incorporating the five variants, calculated as previously described,[13,14] varied among the six patients from 0.063 to 0.725.

Discussion

This study supports the use of WES in the diagnosis and management of lean patients with NAFLD. Two out of 6 patients (33%) with NAFLD without visceral adiposity were discovered to harbour genetic diseases that explain the underlying

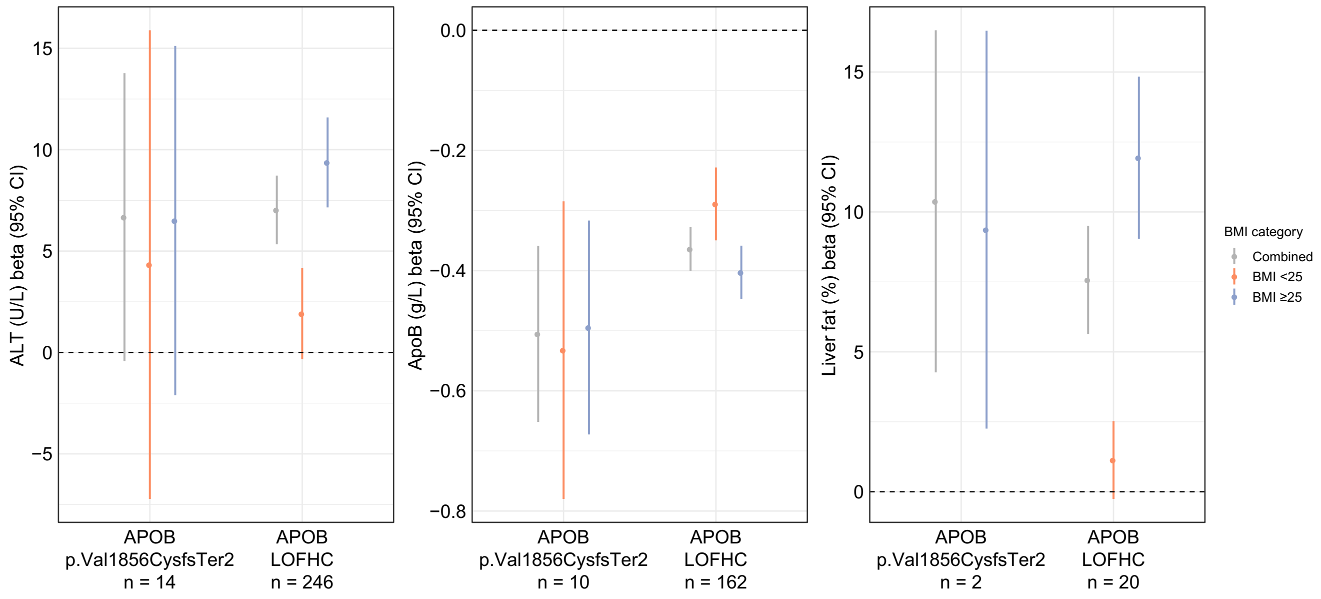

Fig. 1. Effects of LOFHC variants in *APOB* on (A) ALT (B) ApoB levels, and (C) liver fat % in the UK Biobank across BMI strata. Up to 176,177 participants were available for the ALT analysis, up to 175,341 for the ApoB analysis, and up to 18,610 for the liver fat % analysis following quality control and removal of related samples (see Patients and methods). Effect sizes and 95% CIs are reported separately for the p.Val1856CysfsTer2 variant and all other *APOB* LOFHC variants corresponding to linear regression models adjusted for age, sex, and the first 10 principal components of genetic ancestry (liver fat models were additionally adjusted for MRI serial number). We combined all *APOB* LOFHC variants including p.Val1856CysfsTer2 to test for a BMI interaction and noted a significant interaction with both ALT (p = 0.008) and liver fat % ($p = 6.3 \times 10^{-5}$). ALT, alanine aminotransferase; APOB, apolipoprotein B; liver fat %, image-derived liver fat percentage; LOFHC, high-confidence predicted loss-of-function; MRI, magnetic resonance imaging.

pathogenesis of their hepatic steatosis. Furthermore, we validated the pathogenicity of the p.Val1856CysfsTer2 variant in *APOB* using MRI quantification of liver fat and *APOB* levels. In the UK Biobank, we found a significant BMI–rare variant interaction on ALT and liver fat, which suggests that adiposity may amplify the effect of rare variants on fatty liver. This finding parallels what has been previously demonstrated for common variants associated with NAFLD.[15]

In a prior study demonstrating the clinical utility of genomic analysis in the diagnosis and management of adults with unexplained liver disease, three out of six non-obese patients with hepatic steatosis in the absence of metabolic syndrome were found to harbour monogenic disorders underlying the triglyceride accumulation seen on hepatocytes.[4] Subsequently, we have proposed the incorporation of genomic analysis in a variety of liver diseases that remain unexplained despite a comprehensive work-up,[10,16] and in a recent review, we proposed a framework for evaluating patients with lean NAFLD. Although patients with lean NAFLD with increased visceral adiposity likely resemble the broader population with NAFLD, those without visceral adiposity may harbour rare monogenic variants that lead to a phenotype mimicking NAFLD. This study applies the proposed framework[5] to a well-phenotyped cohort of patients with biopsy-proven NAFLD and demonstrates the utility of evaluating patients with lean NAFLD without visceral adiposity for monogenic disorders with a diagnostic yield of 33%. Leveraging the UK Biobank data, we confirmed the pathogenicity of the p.Val1856CysfsTer2 variant in *APOB* and demonstrated an interaction between rare variants in *APOB* and BMI on liver fat and ALT. Heterozygous, rare, predicted loss-of-function variants in *APOB* have been described in other patients with cryptogenic cirrhosis and suggested to contribute to severe disease, including predisposition to hepatocellular carcinoma development.[17–21]

In this study, the two patients with rare monogenic drivers of disease also had common variants in *PNPLA3* and *GCKR*. Prior studies have demonstrated the need to consider the opposing impact of deleterious and protective variants and demonstrated a similar magnitude of opposing effects of variants in *PNPLA3* and *HSD17B13* on MRE.[22] When evaluating polygenic risk, consideration of the combination of common and rare variants may refine our understanding of the risk of NAFLD and fibrosis, as has been described in other diseases including cardiovascular disease and breast cancer.[23]

This prospective, systematic assessment of patients with biopsy-proven lean NAFLD using WES adds new information about pathogenic and actionable rare variants in patients with lean NAFLD. Although the sample size is limited, this study involves well-phenotyped patients with detailed information on liver histology and advanced MRI, which differentiates it from large population-based studies in which most rare variant association studies of NAFLD have been performed. Furthermore, the detailed clinical evaluation allowed for confirmation of genotype–phenotype associations. External validation of the clinical significance of the rare variant in *APOB* in the UK Biobank is an additional strength of the study. APOB deficiency should be suspected in patients with NAFLD in the absence of hyperlipidaemia, in whom circulating APOB levels should be examined.

Unveiling the genetic aetiologies of disease in lean patients with NAFLD may lead to more targeted management, genetic screening of family members, and refined disease prognostication, and potentially uncover actionable pathways for drug development. Furthermore, uncovering the heterogeneous

molecular drivers of NAFLD and fibrosis may improve future clinical trial design by avoiding enrolment of patients with a subtype of disease unlikely to benefit.[16] In conclusion, in this well-characterised cohort of patients with biopsy-proven NAFLD, 33% of patients with lean NAFLD without visceral adiposity harboured monogenic disorders associated with fatty liver, highlighting the value of genetic assessment of NAFLD to identify distinct subtypes of disease.

Abbreviations

APOB, apolipoprotein B; ALT, alanine aminotransferase; FHBL, familial hypobetalipoproteinaemia; LOFHC, high-confidence predicted loss-of-function; MRE, magnetic resonance elastography; MRI, magnetic resonance imaging; MRI-PDFF, magnetic resonance imaging proton density fat fraction; NAFLD, non-alcoholic fatty liver disease; UCSD, University of California San Diego; WES, whole exome sequencing.

Financial support

RL receives funding support from NIEHS (5P42ES010337), NCATS (5UL1TR001442), DOD PRCRP (W81XWH-18-2-0026), NIDDK (U01DK061734, R01DK106419, R01DK121378, R01DK124318, and P30DK120515), NHLBI (P01HL147835), and NIAAA (U01AA029019). VA is supported by NIDDK (K23DK119460). SV receives funding from the NIDDK (K08 DK113109 and R01 DK131033) and Doris Duke Charitable Foundation (2019081).

Conflicts of interest

RL serves as a consultant or advisory board member for Bird Rock Bio, Celgene, Enanta, GRI Bio, Madrigal, Metacrine, NGM, Sanofi, Arrowhead Research, Galmed, NGM, GNI, NovoNordisk, Merck, Siemens, Pfizer, Gilead, and Glympsebio, In addition, his institution has received grant support from Allergan, BMS, BI, Daiichi-Sankyo Inc., Eli-Lilly, Galectin, Galmed, GE, Genfit, Intercept, Janssen Inc, Madrigal, Merck, NGM, Pfizer, Prometheus, Siemens, and Sirius. He is also co-founder of Liponexus Inc. SV serves as a consultant for Albireo Pharma. SA has served as a scientific consultant to Third Rock Ventures. AVK is an employee and holds equity in Verve Therapeutics; has served as a scientific advisor to Amgen, Maze Therapeutics, Navitor Pharmaceuticals, Sarepta Therapeutics, Novartis, Silence Therapeutics, Korro Bio, Veritas International, Color Health, Third Rock Ventures, Illumina, Foresite Labs, and Columbia University (NIH); has received speaking fees from Illumina, MedGenome, Amgen, and the Novartis Institute for Biomedical Research; and has received a sponsored research agreement from IBM Research.

Please refer to the accompanying ICMJE disclosure forms for further details.

Authors' contributions

Study concept and design: RL, SV, VA. Data analysis: MZ, DH, CK, SA, SV. Drafting of the manuscript: MZ, SV, VA. Critical revision and approval of the final manuscript: all authors.

Data availability statement

The datasets generated and/or analysed during the current study are available from the corresponding author on reasonable request in de-identified form.

Supplementary data

Supplementary data to this article can be found online at https://doi.org/10.1016/j.jhepr.2023.100692.

References

Author names in bold designate shared co-first authorship.

[1] Loomba R, Friedman SL, Shulman GI. Mechanisms and disease consequences of nonalcoholic fatty liver disease. Cell 2021;184:2537–2564.

[2] Hagström H, Nasr P, Ekstedt M, Hammar U, Stål P, Hultcrantz R, et al. Risk for development of severe liver disease in lean patients with nonalcoholic fatty liver disease: a long-term follow-up study. Hepatol Commun 2018;2:48–57.

[3] Leung JC, Loong TC, Wei JL, Wong GL, Chan AW, Choi PC, et al. Histological severity and clinical outcomes of nonalcoholic fatty liver disease in nonobese patients. Hepatology 2017;65:54–64.

[4] Hakim A, Zhang X, DeLisle A, Oral EA, Dykas D, Drzewiecki K, et al. Clinical utility of genomic analysis in adults with idiopathic liver disease. J Hepatol 2019;70:1214–1221.

[5] **Vilarinho S**, **Ajmera V**, Zheng M, Loomba R. Emerging role of genomic analysis in clinical evaluation of lean individuals with NAFLD. Hepatology 2021;74:2241–2250.

[6] Tamaki N, Munaganuru N, Jung J, Yonan AQ, Bettencourt R, Ajmera V, et al. Clinical utility of change in nonalcoholic fatty liver disease activity score and change in fibrosis in NAFLD. Clin Gastroenterol Hepatol 2021;19:2673–2674.e3.

[7] Pinheiro FC, Sperb-Ludwig F, Schwartz IVD. Epidemiological aspects of hereditary fructose intolerance: a database study. Hum Mutat 2021;42:1548–1566.

[8] Esposito G, Vitagliano L, Santamaria R, Viola A, Zagari A, Salvatore F. Structural and functional analysis of aldolase B mutants related to hereditary fructose intolerance. FEBS Lett 2002;531:152–156.

[9] Lee J, Hegele RA. Abetalipoproteinemia and homozygous hypobetalipoproteinemia: a framework for diagnosis and management. J Inherit Metab Dis 2014;37:333–339.

[10] Vilarinho S, Mistry PK. Exome sequencing in clinical hepatology. Hepatology 2019;70:2185–2192.

[11] Szustakowski JD, Balasubramanian S, Kvikstad E, Khalid S, Bronson PG, Sasson A, et al. Advancing human genetics research and drug discovery through exome sequencing of the UK Biobank. Nat Genet 2021;53:942–948.

[12] Jurgens SJ, Choi SH, Morrill VN, Chaffin M, Pirruccello JP, Halford JL, et al. Analysis of rare genetic variation underlying cardiometabolic diseases and traits among 200,000 individuals in the UK Biobank. Nat Genet 2022;54:240–250.

[13] Bianco C, Jamialahmadi O, Pelusi S, Baselli G, Dongiovanni P, Zanoni I, et al. Non-invasive stratification of hepatocellular carcinoma risk in non-alcoholic fatty liver using polygenic risk scores. J Hepatol 2021;74:775–782.

[14] Dongiovanni P, Stender S, Pietrelli A, Mancina RM, Cespiati A, Petta S, et al. Causal relationship of hepatic fat with liver damage and insulin resistance in nonalcoholic fatty liver. J Intern Med 2018;283:356–370.

[15] Stender S, Kozlitina J, Nordestgaard BG, Tybjærg-Hansen A, Hobbs HH, Cohen JC. Adiposity amplifies the genetic risk of fatty liver disease conferred by multiple loci. Nat Genet 2017;49:842–847.

[16] Zheng M, Allington G, Vilarinho S. Genomic medicine for liver disease. Hepatology 2022;76:860–868.

[17] Pelusi S, Ronzoni L, Malvestiti F, Bianco C, Marini I, D'Ambrosio R, et al. Clinical exome sequencing for diagnosing severe cryptogenic liver disease in adults: a case series. Liver Int 2022;42:864–870.

[18] Haas ME, Pirruccello JP, Friedman SN, Wang M, Emdin CA, Ajmera VH, et al. Machine learning enables new insights into genetic contributions to liver fat accumulation. Cell Genom 2021;1:100066.

[19] Pelusi S, Baselli G, Pietrelli A, Dongiovanni P, Donati B, McCain MV, et al. Rare pathogenic variants predispose to hepatocellular carcinoma in nonalcoholic fatty liver disease. Sci Rep 2019;9:3682.

[20] Di Filippo M, Moulin P, Roy P, Samson-Bouma ME, Collardeau-Frachon S, Chebel-Dumont S, et al. Homozygous MTTP and APOB mutations may lead to hepatic steatosis and fibrosis despite metabolic differences in congenital hypocholesterolemia. J Hepatol 2014;61:891–902.

[21] Cefalù AB, Pirruccello JP, Noto D, Gabriel S, Valenti V, Gupta N, et al. A novel APOB mutation identified by exome sequencing cosegregates with steatosis, liver cancer, and hypocholesterolemia. Arterioscler Thromb Vasc Biol 2013;33:2021–2025.

[22] Ajmera V, Liu A, Bettencourt R, Dhar D, Richards L, Loomba R. The impact of genetic risk on liver fibrosis in non-alcoholic fatty liver disease as assessed by magnetic resonance elastography. Aliment Pharmacol Ther 2021;54:68–77.

[23] Khera AV, Chaffin M, Aragam KG, Haas ME, Roselli C, Choi SH, et al. Genome-wide polygenic scores for common diseases identify individuals with risk equivalent to monogenic mutations. Nat Genet 2018;50:1219–1224.

Journal of Hepatology, Volume ■

Supplemental information

Genomic analysis of lean individuals with NAFLD identifies monogenic disorders in a prospective cohort study

Melanie Zheng, Daniel Q. Huang, Chigoziri Konkwo, Saaket Agrawal, Amit V. Khera, Rohit Loomba, Sílvia Vilarinho, and Veeral Ajmera

Genomic analysis of lean individuals with NAFLD identifies monogenic disorders in a prospective cohort study

Melanie Zheng, Daniel Huang, Chigoziri Konkwo, Saaket Agrawal, Amit V. Khera, Rohit Loomba, Sílvia Vilarinho, Veeral Ajmera

Table of contents

Supplementary methods..2

Fig. S1..5

Table S1..6

Table S2..7

Table S3..8

Table S4..9

Table S5..10

Table S6..11

Supplementary references..12

Supplementary methods

Inclusion and Exclusion Criteria

Participants meeting any of the following criteria were excluded from the study: significant alcohol consumption (defined as ≥14 drinks/week for men or ≥7 drinks/week for women) within the previous 2-year period; evidence of active substance use. Alcohol intake history was obtained in a clinical setting and verified at the research clinic with the Alcohol Use Disorders Identification Test and the Skinner questionnaire. Other causes of liver disease and hepatic steatosis were ruled out systematically based on history and laboratory tests. Participants were instructed to fast for a minimum of eight hours before collection of laboratory tests.

Whole-exome sequencing and analysis

Germline DNA was extracted from blood samples using standard methods. Germline DNA was captured using IDT xGen exome V2 exome enrichment probes and sequenced using the Illumina NovaSeq platform. Exome sequencing data were mapped and aligned to the reference human genome build 19 using Burrows-Wheeler Aligner.(1) Variants were called using GATK(2) and annotated using Annovar.(3) All variants passed an initial quality control and were filtered out for read depth of coverage < 30 and for segmental duplications (Figure 1). Protein-altering variants were selected by removing synonymous and intronic/non-coding variants. Variants were selected for minor allele frequency (MAF) of <0.01 for homozygous and compound heterozygous variants (recessive inheritance) or $<2 \times 10^{-5}$ for heterozygous variants (dominant inheritance). MAF was determined using the genome aggregation database (gnomAD).(4) Variants were then prioritized based on predicted deleteriousness, using

Combined Annotation Dependent Depletion (CADD)(5) score > 20 for missense variants and SpliceAI(6) score > 0.5 for splice-site variants. Remaining variants were flagged based on an internal list of 264 liver disease-related genes derived from Online Mendelian Inheritance in Man (OMIM) database entries, previously described.(7) Selected NAFLD-associated polymorphisms, namely *PNPLA3* rs738409:p.I148M, *GCKR* rs1260326:p.P446L, *TM6SF2* rs58542926:C/T, and *HSD17B13* rs72613567:T/TA, MBOAT7-TMC4 rs641738:C/T were extracted from WES data.

UK Biobank Cohort and Analysis

Phenotypes

The UK Biobank is an observational study that enrolled over 500,000 individuals between the ages of 40 and 69 years between 2006 and 2010(8). Alanine aminotransferase (UKB field 30620) and apolipoprotein B (UKB field 30640) measured at the time of enrollment were made available to researchers. Imaging-derived liver fat was derived in 36,703 participants of the UK Biobank as previously described(9). This analysis of data from the UK Biobank was approved by the Mass General Brigham institutional review board and was performed under UK Biobank application #7089.

APOB rare variant validation

We conducted rare variant association studies using the first 200,643 exomes from the UK Biobank.(10) An extensive quality control procedure was applied to these data prior to analysis as described elsewhere (11). Following quality control, 200,337 exomes were available for analysis. To identify rare (minor allele frequency < 0.1%)

high-confidence predicted inactivating variants in *APOB*, we applied the previously validated Loss-Of-Function Transcript Effect Estimator (LOFTEE) algorithm implemented within the Ensembl Variant Effect Predictor (VEP) software program as a plugin, VEP version 96.0 (4). We refer to these variants are "LOFHC".

Statistical analysis

All effect sizes are reported from linear regressions adjusted for age (at enrollment for ALT and APOB, at the time of imaging for liver fat %), sex, and the first 10 principal components of genetic ancestry (liver fat analyses were additionally adjusted for MRI serial number). Prior to analysis, one sample of a pair was randomly excluded if that pair had second-degree relative or closer kinship. Carrier counts in Figure 2 are reported following this exclusion and correspond to participants with the studied phenotype available. Analyses were repeated in low (< 25 kg/m^2) and high (>= 25 kg/m^2) body mass index (BMI) subgroups. The interaction between *APOB* rare variant carrier status and BMI was tested using a linear regression including BMI and a carrier status by BMI interaction term along with the above covariates. All analyses were performed using R 3.6.0.

Fig. S1. Whole exome sequencing variant filtering pipeline revealing the pathogenic mutation in *ALDOB* for Patient 1 and *APOB* for Patient 2. MAF, minor allele frequency; CADD, Combined Annotation Dependent Depletion; OMIM, Online Mendelian Inheritance in Man.
*list from Zheng M, Allington G, Vilarinho S. Genomic medicine for liver disease. *Hepatology.* 2022.

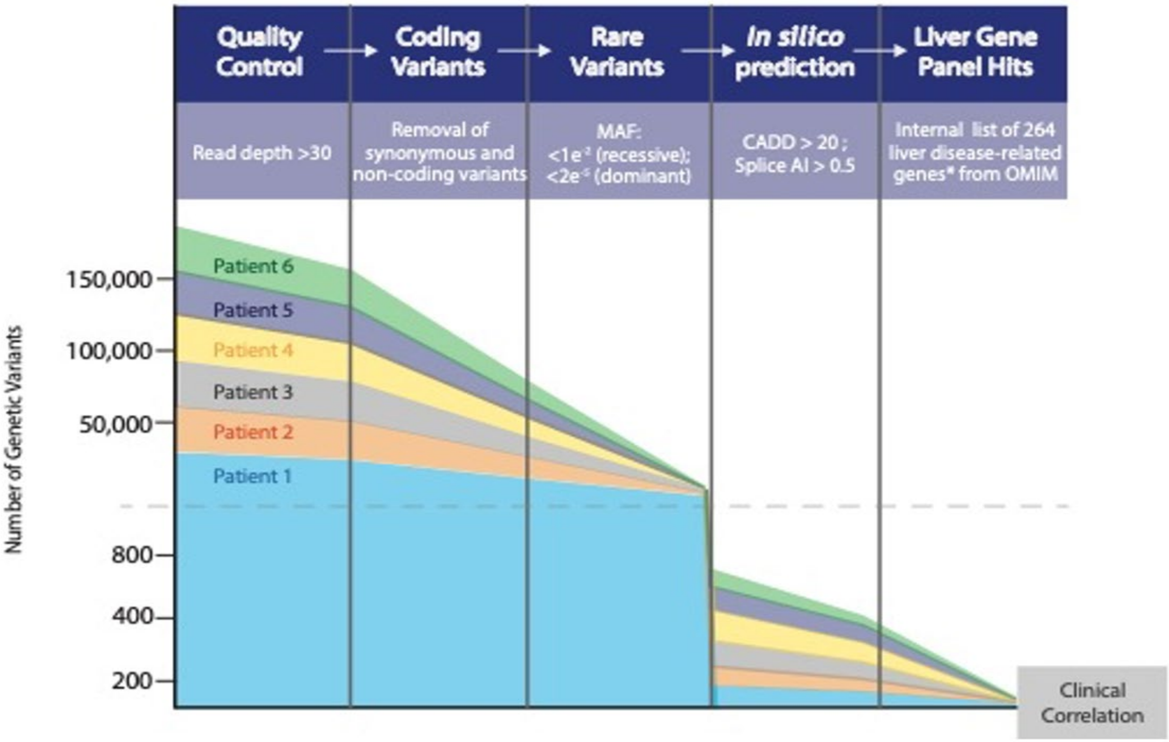

Table S1. Baseline characteristics of participants with NAFLD, stratified by baseline body mass index

	**Overall** (N=124)	**Non-lean** (N=118)	**Lean** (N=6)	**P value**
**Demographic profile**				
Age (yr)	57.00 [47.75, 65.00]	57.00 [47.25, 65.00]	59.50 [53.75, 63.00]	0.834
Female, n (%)	78 (62.9)	73 (61.9)	5 (83.3)	0.529
BMI (kg/m^2)	31.82 [29.00, 36.53]	32.24 [29.40, 36.90]	23.42 [21.74, 24.54]	<0.001
Diabetes Mellitus, n (%)	61 (49.2)	58 (49.2)	3 (50.0)	1
Hispanic, n (%)	48 (38.7)	47 (39.8)	1 (16.7)	0.48
**Biochemical data**				
AST (u/L)	35.00 [26.00, 59.00]	35.00 [26.00, 58.75]	41.50 [36.50, 72.75]	0.292
ALT (u/L)	46.50 [32.00, 78.00]	46.50 [32.00, 77.50]	45.50 [43.25, 120.50]	0.386
HbA1c (%)	6.00 [5.60, 6.70]	6.00 [5.60, 6.70]	5.95 [5.67, 6.15]	0.834
Total Bilirubin (mg/dl)	0.49 [0.30, 0.60]	0.49 [0.30, 0.60]	0.50 [0.35, 0.65]	0.787
Direct Bilirubin (mg/dl)	0.10 [0.10, 0.19]	0.10 [0.10, 0.19]	0.15 [0.10, 0.20]	0.963
INR	1.00 [1.00, 1.10]	1.00 [1.00, 1.10]	1.05 [1.00, 1.10]	0.516
Albumin (g/dl)	4.40 [4.30, 4.60]	4.40 [4.30, 4.60]	4.70 [4.55, 4.77]	0.027
Total cholesterol (mg/dL)	182.50 [156.75, 207.50]	182.50 [156.25, 206.75]	188.00 [167.00, 215.00]	0.692
HDL (mg/dL)	43.00 [36.00, 54.25]	43.00 [36.00, 54.00]	46.00 [40.50, 56.75]	0.552
LDL (mg/dL)	100.50 [82.75, 124.50]	100.50 [82.25, 125.50]	110.00 [90.25, 120.75]	0.847
TG (mg/dL)	142.00 [110.00, 196.25]	142.00 [110.25, 196.00]	149.50 [103.50, 191.00]	0.912
Interval between biopsies (months)	17.85 [8.47, 32.62]	17.85 [8.43, 33.47]	17.35 [13.53, 24.18]	0.87
**Liver histology findings baseline**				
NAS	5.00 [4.00, 6.00]	5.00 [4.00, 6.00]	5.50 [4.25, 6.00]	0.7
Fibrosis stage, n (%)				
0	34 (27.4)	33 (28.0)	1 (16.7)	0.59
1	45 (36.3)	44 (37.3)	1 (16.7)	
2	15 (12.1)	14 (11.9)	1 (16.7)	
3	19 (15.3)	17 (14.4)	2 (33.3)	
4	11 (8.9)	10 (8.5)	1 (16.7)	
Steatosis score, n (%)				
0	1 (0.8)	1 (0.8)	0 (0.0)	0.838
1	41 (33.1)	40 (33.9)	1 (16.7)	
2	49 (39.5)	46 (39.0)	3 (50.0)	
3	33 (26.6)	31 (26.3)	2 (33.3)	
Lobular inflammation score, n (%)				
0	0	0	0	0.793
1	45 (36.6)	43 (36.8)	2 (33.3)	
2	71 (57.7)	67 (57.3)	4 (66.7)	
3	7 (5.7)	7 (6.0)	0 (0.0)	
Ballooning score, n (%)				
0	12 (9.7)	11 (9.3)	1 (16.7)	0.833
1	70 (56.5)	67 (56.8)	3 (50.0)	
2	42 (33.9)	40 (33.9)	2 (33.3)	
**Imaging results**				
MRI-PDFF (%)	14.05 [8.69, 19.50]	13.90 [8.62, 19.35]	18.89 [14.57, 23.40]	0.179
MR elastography	2.92 [2.50, 3.89]	2.92 [2.50, 3.88]	3.03 [2.82, 3.70]	0.936

Median values are provided with IQR in parenthesis, unless otherwise noted as n (%)
Abbreviations: NAS, NAFLD activity score; yr, year; SD, standard deviation; AST, aspartate transaminase; ALT, alanine transaminase; HbA1c, hemoglobin A1c; MRI-PDFF, magnetic resonance imaging – proton density fat fraction
Categorical variables tested with chi square.
Continuous variables compared using t-test or Wilcoxon two-sample test as appropriate.

Table S2. Change in fibrosis stage in participants with NAFLD, stratified by body mass index

	**Non-lean** (N=118)	**Lean** (N=6)	p-value
**Change in fibrosis stage**			
-2	2 (1.7)	0 (0.0)	0.413
-1	23 (19.5)	0 (0.0)	
0	63 (53.4)	6 (100.0)	
1	25 (21.2)	0 (0.0)	
2	4 (3.4)	0 (0.0)	
3	1 (0.8)	0 (0.0)	
**Fibrosis progression**	30 (25.4)	0 (0.0)	0.352
**Fibrosis regression**	25 (21.2)	0 (0.0)	0.459
**Change in NAS**	-1.00 [-2.00, 0.00]	-1.50 [-2.00, -1.00]	0.495
**Change in MRE (kPa)**	-0.14 [-0.54, 0.32]	-0.12 [-0.16, -0.12]	0.707
**Change in MRI-PDFF**	-1.17 [-4.03, 1.70]	-4.00 [-4.10, -1.75]	0.521

Values reported as n (%)

Table S3: Whole-exome sequencing metrics of the lean patient cohort with NAFLD (n=6).

Variable	Mean	Range
Mean depth (x)	67.1	(63.4 - 70.1)
% Error rate	0.358	(0.34 - 0.38)
% of targeted bases >8 independent reads	98	(98.0 - 98.0)
% of targeted bases >30 independent reads	94.8	(94.1 - 95.5)

Table S4: Table 2. Summary of genotype-phenotype data for the two adult lean patients with type 2 NAFLD who were found to have monogenic diagnoses.

Genotype Information									
**Patient ID**	Affected Gene	Zygosity	AA Mutation/ Consequence	gnomAD MAF (overall)	gnomAD MAF (max sub-population)	CADD score	MetaSVM score	**Clinical Phenotype**	**Genetic Diagnosis**
1	*ALDOB*	Homozygous	p.Ala150Pro	$3.093e^{-3}$	$4.866e^{-3}$ (non-Finnish European)	31	0.511	NAFLD type 2	Hereditary fructose intolerance
2	*APOB*	Heterozygous	p.Val1856CysfsTer2	$3.979e^{-6}$	$6.153e^{-5}$ (African)	N/A	N/A		Familial hypobetalipoproteinemia

AA, amino acid; gnomAD, Genome Aggregation Database; MAF, minor allele frequency; CADD, Combined Annotation Dependent Depletion; MetaSVM, Meta-analytic support vector machine; N/A, not applicable

Table S5: Enrichment of LOFHC variants in *APOB*, *MTTP*, and *TM6SF2* in UK Biobank participants with low apolipoprotein B

	Genotype Information				
Trait	Variant Set	Case Frequency (%)	Control Frequency (%)	Odds Ratio (95% CI)	P-value
Bottom 10% apolipoprotein B (apoB <= 0.74 g/L)	*APOB* LOFHC	130/17,424 (0.75%)	42/157,917 (0.03%)	29.2 (20.6 – 41.4)	6.0×10^{-95}
	MTTP LOFHC	24/17,424 (0.14%)	105/157,917 (0.07%)	2.1 (1.4 – 3.3)	1.2×10^{-3}
	TM6SF2 LOFHC	10/17,424 (0.06%)	56/157,917 (0.04%)	1.7 (0.9 – 3.3)	0.16

Participants were dichotomized by whether they were in the bottom decile of apolipoprotein B in the UK Biobank. Among the 200,337 UK Biobank exomes following quality control, 294 participants harbored one of 105 LOFTEE-derived high confidence predicted loss-of function (LOFHC) variants in *APOB* (including the p.Val1856CysfsTer2 variant discussed in the main text), 145 participants harbored one of 29 LOFHC variants in *MTTP*, and 75 participants harbored one of 17 LOFHC variants in *TM6SF2*. Following quality control and removal of related samples (see Methods), 175,341 UK Biobank participants were available for the present analysis. Effect sizes and standard errors used to generate 95% confidence intervals were obtained from Firth logistic regression, while p-values were obtained from the SPA test, both as implemented in the R package SPAtest. Models were adjusted for age, sex, and the first ten principal components of genetic ancestry. Note that the number of carriers listed in the table is fewer than those reported in the legend because of (1) removal of related samples prior to analysis and (2) several rare variant carriers having missing apolipoprotein B.

Table S6: Evaluation of common variants and polygenic risk score associated with NAFLD and fibrosis in lean NAFLD patients

Patient ID	Genotype Information					Polygenic Risk Score*
	PNPLA3 rs738409:p.I148M	GCKR rs1260326:p.P446L	TM6SF2 rs58542926:C/T	HSD17B13 rs72613567:T/TA	MBOAT7-TMC4 rs641738:C/T	
1	Wild-type	Heterozygous	Wild-type	Wild-type	Heterozygous	0.128
2	Homozygous	Heterozygous	Wild-type	Wild-type	Heterozygous	0.66
3	Wild-type	Wild-type	Wild-type	Wild-type	Heterozygous	0.063
4	Heterozygous	Heterozygous	Wild-type	Wild-type	Heterozygous	0.394
5	Heterozygous	Wild-type	Wild-type	Wild-type	Homozygous	0.392
6	Homozygous	Homozygous	Wild-type	Wild-type	Heterozygous	0.725

*Polygenic risk score calculated as previously described, 0.266*PNPLA3 + 0.274*TM6SF2 + 0.065*GCKR + 0.063*MBOAT7 – 0.361*HSD17B13 (12, 13)

Supplementary references

1. Li H, Durbin R. Fast and accurate short read alignment with Burrows-Wheeler transform. Bioinformatics 2009;25:1754-1760.
2. Van der Auwera GA, Carneiro MO, Hartl C, Poplin R, del Angel G, Levy-Moonshine A, Jordan T, et al. From FastQ Data to High-Confidence Variant Calls: The Genome Analysis Toolkit Best Practices Pipeline. Current Protocols in Bioinformatics 2013;43:11.10.11-11.10.33.
3. Wang K, Li M, Hakonarson H. ANNOVAR: functional annotation of genetic variants from high-throughput sequencing data. Nucleic Acids Research 2010;38:e164-e164.
4. Karczewski KJ, Francioli LC, Tiao G, Cummings BB, Alföldi J, Wang Q, Collins RL, et al. The mutational constraint spectrum quantified from variation in 141,456 humans. Nature 2020;581:434-443.
5. Rentzsch P, Witten D, Cooper GM, Shendure J, Kircher M. CADD: predicting the deleteriousness of variants throughout the human genome. Nucleic Acids Research 2019;47:D886-D894.
6. Jaganathan K, Kyriazopoulou Panagiotopoulou S, McRae JF, Darbandi SF, Knowles D, Li YI, Kosmicki JA, et al. Predicting Splicing from Primary Sequence with Deep Learning. Cell 2019;176:535-548.e524.
7. Zheng M, Allington G, Vilarinho S. Genomic medicine for liver disease. Hepatology 2022;76:860-868.
8. Sudlow C, Gallacher J, Allen N, Beral V, Burton P, Danesh J, Downey P, et al. UK biobank: an open access resource for identifying the causes of a wide range of complex diseases of middle and old age. PLoS Med 2015;12:e1001779.
9. Haas ME, Pirruccello JP, Friedman SN, Wang M, Emdin CA, Ajmera VH, Simon TG, et al. Machine learning enables new insights into genetic contributions to liver fat accumulation. Cell Genom 2021;1.
10. Szustakowski JD, Balasubramanian S, Kvikstad E, Khalid S, Bronson PG, Sasson A, Wong E, et al. Advancing human genetics research and drug discovery through exome sequencing of the UK Biobank. Nat Genet 2021;53:942-948.
11. Jurgens SJ, Choi SH, Morrill VN, Chaffin M, Pirruccello JP, Halford JL, Weng LC, et al. Analysis of rare genetic variation underlying cardiometabolic diseases and traits among 200,000 individuals in the UK Biobank. Nat Genet 2022;54:240-250.
12. Dongiovanni P, Stender S, Pietrelli A, Mancina RM, Cespiati A, Petta S, Pelusi S, et al. Causal relationship of hepatic fat with liver damage and insulin resistance in nonalcoholic fatty liver. J Intern Med 2018;283:356-370.
13. Bianco C, Jamialahmadi O, Pelusi S, Baselli G, Dongiovanni P, Zanoni I, Santoro L, et al. Non-invasive stratification of hepatocellular carcinoma risk in non-alcoholic fatty liver using polygenic risk scores. J Hepatol 2021;74:775-782.
